# Connectivity Benefits Most Woodland Invertebrate Species but Only in Landscapes With Low Woodland Cover

**DOI:** 10.1111/ele.70131

**Published:** 2025-05-19

**Authors:** Charles A. Cunningham, Colin M. Beale, Diana E. Bowler, Michael J. O. Pocock, Robin Hutchinson, Piran C. L. White, Merryn Hunt, Lindsay Maskell, Jane K. Hill

**Affiliations:** ^1^ Department of Biology University of York York UK; ^2^ Leverhulme Centre for Anthropocene Biodiversity University of York York UK; ^3^ York Environmental Sustainability Institute University of York York UK; ^4^ UK Centre for Ecology & Hydrology Oxfordshire UK; ^5^ Department of Environment and Geography University of York York UK; ^6^ UK Centre for Ecology & Hydrology, Lancaster Environment Centre Bailrigg UK

**Keywords:** circuit‐model, citizen science, fragmentation, habitat creation, inlabru, recovery, resilience, spatial prioritisation, species occurrence, treescape

## Abstract

Connectivity is widely assumed to benefit biodiversity, but this has not been extensively quantified across multiple taxa and landscapes. Focusing on the UK, where woodland cover is low (13%), we analysed species occurrence records from citizen science for over 800 broadleaf woodland‐associated invertebrate species from 15 taxonomic groups in relation to woodland cover and connectivity. Overall, increased woodland connectivity positively affects broadleaf‐associated species occurrence (effect of connectivity across species, accounting for positive effect of broadleaf cover). The benefits of connectivity varied considerably by species: 39% of species showed a significant positive effect, while for 3% it was significantly negative. However, the interaction between cover and connectivity revealed that, overall, connectivity benefits are only found in low cover landscapes. Our findings emphasise potential biodiversity benefits from maximising connectivity when increasing woodland cover and highlight the importance of spatial targeting in restoration efforts, especially in landscapes with low woodland cover.

## Graphical abstract

Connectivity is widely assumed to benefit biodiversity, but this has not been extensively quantified across multiple taxa and landscapes. Using species occurrence records from citizen science for over 800 broadleaf‐associated species in the UK, we found an overall positive effect of woodland connectivity, despite large between‐species variance. Our results especially emphasise the importance of connectivity for landscapes with low woodland cover.

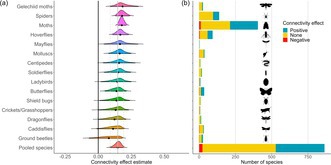

## 1 Introduction

The Anthropocene world is experiencing a period of rapid ecological change, making it crucial to mitigate detrimental impacts to ensure a sustainable future (Jaureguiberry et al. 2022; Pereira et al. 2024; Secretariat of the Convention on Biological Diversity 2020). The Kunming‐Montreal Global Biodiversity Framework sets 23 global targets to protect and restore ecosystems, including restoring 30% of all degraded ecosystems and conserving 30% of land, waters and seas (CBD 2022). To meet these biodiversity targets, improving connectivity is considered to be an important ecological principle (Watson et al. 2023). Although landscape‐scale land use is known to shape local faunal communities and species trends (Le Provost et al. 2021; Seibold et al. 2019), there is a paucity of empirical studies that quantify the positive effects of landscape connectivity beyond a few terrestrial species. Connectivity is increasingly being incorporated into policy and conservation strategy (CBD 2022; Hordley et al. 2022; Lawton et al. 2010), and so studies that improve understanding of whether connectivity has benefits for biodiversity across a wide range of taxa are vital.

Connectivity is usually considered to have a positive effect on biodiversity by allowing individuals to move more easily through fragmented landscapes, thereby increasing occupancy in fragments and gene flow across the population, leading to larger, more persistent populations (Beger et al. 2022). Studies of spatial configuration of habitat can also be framed as fragmentation in the negative context of habitat loss (de Albuquerque and Rueda 2010; Wang et al. 2014), and hence increasing connectivity is a way to mitigate or reverse the negative impacts of fragmentation (Humphrey et al. 2015) especially in areas such as Europe, with low coverage of wildlife‐rich habitat. However, we know less about these potential positive effects (Fletcher et al. 2016) and the relative importance of connectivity/fragmentation in contrast to habitat availability is still debated (Fahrig et al. 2019; Fletcher et al. 2018; Riva, Haddad, et al. 2024; Riva et al. 2025). In order to incorporate connectivity into effective habitat restoration planning, understanding connectivity benefits beyond individual species or selected taxa is vital as species use landscapes in different ways (Savary et al. 2024).

Woodlands (we use ‘woodland’ rather than ‘forest’ to describe tree cover as this is more typically used in the UK context) provide many ecosystem services and biodiversity benefits (Burton et al. 2018), including sequestering carbon and shaping the composition of biological communities (Bowler et al. 2023; Daskalova et al. 2020). As well as protecting existing woodlands, the creation of new woodland is needed in many countries to meet net zero emission commitments and ecosystem restoration targets in the face of intensifying climate change (Bateman et al. 2022; Luby et al. 2022). Where this additional woodland should be created is contentious because the benefits and costs vary spatially and are altered by current woodland characteristics, such as species composition (Sandoval‐Martínez et al. 2023), tree density (Sanczuk et al. 2023), and woodland spatial configuration (Savary et al. 2024). Evidence from previous empirical studies has shown that woodland established closer to existing woodland grows faster and has greater structural diversity (Hughes et al. 2023a), and that species can more rapidly colonise this new habitat (Hughes et al. 2023b). Greater connectivity can also facilitate climate‐driven range shifts of species (Hodgson et al. 2022). However, connectivity can also have potentially negative ecological effects, for example facilitating the successful establishment and spread of pests, pathogens or invasive species (Maguire et al. 2015) although evidence for this is limited (Haddad et al. 2014). Overall, the benefits of woodland connectivity for biodiversity are still uncertain (Bailey 2007), requiring studies that compare a wide range of species and taxa (Bowler et al. 2023).

We focus our study on the UK because of the availability of extensive biological record datasets spanning many taxonomic groups (Outhwaite et al. 2020). These datasets are curated and verified through different recording schemes, each focusing on a specific taxonomic group, but together enable robust analyses of multiple taxa, including invertebrates that are often overlooked in studies focusing on iconic vertebrate groups such as birds and mammals despite having an important role in ecosystems (Chowdhury et al. 2023). The UK has low woodland coverage, although it has increased from approximately 4.7% land cover at the beginning of the 20th century to 13% today (Forest Research 2023), with a statutory target to reach 16.5% tree and woodland cover by 2050 (DEFRA 2023). This increase was primarily driven by the establishment of commercial conifer plantations in the 20th century, and more recently by broadleaf tree planting (Raum 2020), but nonetheless the UK remains one of the least wooded countries in Europe (Liu et al. 2023) and so questions about connectivity benefits are particularly pertinent.

Here, we quantify the ecological benefits of woodland connectivity by analysing data from 3277 species from 15 invertebrate recording schemes, of which 864 species were found to be broadleaf‐associated. We quantify woodland cover and connectivity across the UK (1 km^2^ resolution) and relate these to local species occurrences (18 million records collected by volunteer recorders). We (1) test the hypothesis that woodland connectivity is positively associated with species occurrence, after accounting for the effects of woodland cover; and (2) examine how the effects of connectivity vary between species. We (3) examine interactions between cover and connectivity and their predicted effect on species occurrence.

## 2 Material and Methods

### 2.1 Species Occurrence Data

We analysed unstructured species occurrence records for 3277 species from 15 recording schemes, each of which curate citizen science recording of specific taxonomic groups, covering butterflies and moths (Lepidoptera), caddisflies (Trichoptera), centipedes (Chilopoda), crickets/grasshoppers (Orthoptera), dragonflies and damselflies (Odonata), gelechiid moths (Lepidoptera: Gelechiidae), ground beetles (Coleoptera: Carabidae), hoverflies (Diptera: Syrphidae), ladybirds (Coleoptera: Coccinellidae), mayflies (Ephemeroptera), molluscs (Mollusca), shield bugs (Hemiptera: Pentatomoidea), soldierflies (Diptera: Stratiomyidae) and allies and spiders (Arachnida: Araneae and Opiliones) (see Table S1 for numbers of species, records, and associated UK recording schemes). Recording schemes were selected which included records that represented the entire UK and had records available up until at least 2018 (see Supporting Information: Methods A for further details).

Each occurrence record represents an observation of a specific species at a particular place and time. We only included records with a precise date (day, month and year) and location (1 km Ordnance Survey grid cell resolution or finer). The data from each recording scheme were analysed separately using a standardised workflow. We structured the occurrence records into binary detection–non‐detection (i.e., species presence detected, not detected) data (Isaac et al. 2014). A ‘visit’ was defined as a list of one or more species recorded in a 1 km Ordnance Survey grid square on a single date. For a given visit, the species recorded were classed as detected, value of 1; and species not recorded for that visit, but recorded on other visits for the same recording scheme, were given a non‐detection value of 0 for that visit.

Our datasets of species occurrences span several decades during which species ranges and occurrences have changed, as well as woodland cover and connectivity. In our analyses, we account for these temporal changes by computing presence‐absence information for two time periods. We consider species data from 1990–1999 and 2015–2021 (exact dates vary by recording scheme, which had different periods available at time of data request), subsequently termed 1990 and 2015 periods, comprising about 6 million and 12 million species records across the 15 recording schemes, respectively (Table S1). These dates were chosen to broadly align with the cover and connectivity data available from Land Cover Maps (see below). Species records from before 1990 and 2000–2014 inclusive were therefore removed to create distinct occurrence and woodland time windows, thereby accounting for woodland cover and connectivity changes. The Orkney and Shetland island groups were excluded from the analysis due to extremely low tree cover on these islands, as were any other islands smaller than 10 km^2^.

### 2.2 Woodland Cover and Climate Variables

We extracted UK broadleaf woodland and coniferous woodland cover data separately using UK Land Cover Maps (25 m grid resolution datasets; for England, Scotland, Wales and Northern Ireland) for 1990 and 2015 (Rowland et al. 2017, 2020). These maps classify ‘broadleaf woodland’ as stands taller than 5 m with > 20% cover, or > 30% scrubland (i.e., stands below 5 m). ‘Coniferous woodland’ encompasses semi‐natural stands and commercial plantations with a tree cover > 20% and primarily composed of coniferous species, including felled areas assumed likely to be replanted. We used this information to create maps of broadleaf and coniferous woodland presence/absence (25 m resolution).

To account for other factors that may affect the spatial variation in the occurrences of woodland species in later modelling, we also created five ecologically relevant climate variables considered likely to affect insect population dynamics either directly (e.g., physiological limits) or indirectly (i.e., impacting habitat, food or host plants), similar to variables used in Palmer et al. (2017). These included growing degree days above 5°C; mean temperature of the coldest month; coefficient of variation in daily temperature (K), that is, seasonality; annual precipitation; and soil moisture. The first four of these variables were calculated using the HADUK‐Grid Gridded Climate Observations on a 1 km grid over the UK (Met Office et al. 2022). Soil moisture was included from Grid‐to‐Grid model estimates of soil moisture for Great Britain and Northern Ireland on a 1 km grid produced as part of UK‐SCAPE (Kay et al. 2021). We calculated climate variables for the two time periods (1990, 2015) to match the woodland cover and species occurrence data, by aggregating using the mean annual value for different annual groupings (1 km grid resolution); 1980–1999 for the 1990 period, and 2000–2019 for the 2015 period (apart from soil moisture where data were available only for years 1981–1999 and 2000–2010).

### 2.3 Quantifying Connectivity

Connectivity can be measured in many different ways (Beger et al. 2022; Brückmann et al. 2010), including structural connectivity (e.g., FragStats), potential functional connectivity (e.g., least‐cost path or circuit model approaches), or patch‐based network analysis (Fletcher and Fortin 2018). Given both the 1 km grid resolution of the records and the large number of species included which will use landscapes in different ways, a landscape, rather than patch‐based, approach to quantifying connectivity was most appropriate (Moilanen 2011; Dennis, Huck, Holt and McHenry 2024). In order to calculate a single measure of potential functional connectivity, we aggregated broadleaf and coniferous woodland maps into a single layer mapping woodland structure, and calculated woodland connectivity from this separately for 1990 and 2015. We computed a single landscape connectivity metric because species‐specific information on dispersal and other movement‐related life history traits is lacking for almost all of the species included in our analysis. An overview of the methodological work flow is provided in Figure S1.

We used the *Omniscape* circuit‐model approach (Landau et al. 2021; McRae et al. 2016) that has been widely used to simulate potential functional connectivity (Belote et al. 2022; Martinez‐Cillero et al. 2023; Suraci et al. 2023) to compute connectivity for each 25 m pixel. *Omniscape* simulates the movement of organisms across a landscape as the flow of electrical current through a circuit. The algorithm uses a moving window approach, iteratively treating every individual 25 m woodland pixel as a target for electrical current and connecting that pixel to all other woodland pixels (source cells) within a given radius. The metric is affected by both cover and fragmentation, so we account for woodland cover independently of connectivity in our later modelling stage. See Landau et al. (2021) for a fuller description of the *Omniscape* approach.

In our study, we calculated connectivity using each 25 m raster pixel, that is, at the same resolution as the Land Cover Maps. We used a buffer distance of 4 km around focal 25 m target ground pixels to represent landscape‐scale connectivity. We set a single resistance value of 100 for all non‐woodland pixels (i.e., assuming species find it 100 times more difficult to move through non‐woodland pixels). This value minimised cover‐connectivity correlation, which is higher with smaller resistance values; whilst also reflecting evidence of non‐woodland matrix permeability for UK woodland species (Eycott et al. 2011). To explore the consequences of these decisions, we tested different buffer distances and resistance values (see Supporting Information: Methods B). As these were highly correlated with the selected connectivity layer of 4 km radius and 100 resistance, we considered them unlikely to qualitatively affect our conclusions and so we do not discuss them further in the main text (Figure S2).

We transformed the 25 m datasets to a 1 km grid of woodland cover (proportion cover in each 1 km^2^) and connectivity (median within each 1 km^2^) to align with the resolution of species, climate and soil data.

### 2.4 Exploring Species Associations With Connectivity

We modelled the probability of species occurrence on a visit and its dependence on woodland cover and connectivity (1990 and 2015) at a 1 km grid resolution for the UK. We used the *inlabru* R package (Bachl et al. 2019; Bakka et al. 2018; Morera‐Pujol et al. 2022; Seaton et al. 2024) to create a Bayesian spatio‐temporal model of occurrence probability for each species (i.e., 3277 separate models). For all species we included broadleaf woodland cover, coniferous woodland cover, and total connectivity, as well as broadleaf cover:connectivity and coniferous cover:connectivity interactions as our fixed effects. See Figures S3–S5 for cover and connectivity values for both periods, and change between them.

A 2D‐mesh was created to cover the UK over which a Matérn stochastic partial differential equation (SPDE) was fitted to represent spatial autocorrelation. By additionally including an AR(1) autoregressive random effect between the two time periods, this also allowed us to model temporal autocorrelation (Wiethase et al. 2024). Fitting our models over two linked time periods means that we provide a strong test of all covariate associations: with changes in both occurrence and covariate patterns over time, only associations that remain across both time periods will be identified by our models, reducing the risk of identifying spurious spatial correlations.

We also included the five climate variables in the model as second‐order random walk effects (Gómez‐Rubio 2020; Rue and Held 2005) to allow for non‐linear relationships (growing degree days above 5°C, mean temperature of the coldest month, coefficient of variation in daily temperature, annual precipitation, and soil moisture; variable correlation Figure S6). All climate variables, along with cover and connectivity, were standardised to have a mean of 0 and a standard deviation of 1.

As we used unstructured species occurrence data, our data could be affected by sampling variability. We accounted for species phenologies and variation in visit dates among sites and years by including week of year as a cyclic second order random walk term effect which allowed for flexible fitting of highly variable species phenologies, that is, unimodal, bimodal etc. We also included list length, defined as the number of species recorded on a given visit for a given recording scheme, as a measure of the recorder effort, included as a fixed effect factor with three levels: single record (1 species), short visit (2–3 species), or long visit (4+ species) (Bowler et al. 2021; Boyd et al. 2023). See Supporting Information: Methods C for model formula.

### 2.5 Testing the Importance of Connectivity Using Meta‐Analysis

We used meta‐analysis to combine the species‐level estimates and explore the general benefits of connectivity among recording schemes. Of the 3277 modelled species, the models of 2271 species converged, and so these were taken forward as they provided estimated effect sizes for the meta‐analysis.

We separated out broadleaf woodland‐associated species using the broadleaf cover effect from the modelling stage. Of the converged species models, 864 species showed a broadleaf association (38.0%), which was assessed by whether the effect of broadleaf woodland cover on occurrence had a 95% credible interval entirely above zero. In the main text, we focus analyses only on broadleaf‐associated species because broadleaf woodland comprises the vast majority of current native and planned UK woodland cover, but as a comparison, we also defined two other woodland association groups: coniferous‐associated (with credible interval of the coniferous cover effect above 0 = 508 species) and woodland‐avoiding species (with credible interval of both broadleaf and coniferous cover below zero = 119 species). We present outputs for the other two woodland association groups in the Supporting Information (Figures S7 and S8) but do not present these in the main results. There were 345 species that were associated with both broadleaf and coniferous woodland (and hence included in both analyses), and 1125 species that did not fall into any of the three association groups above.

To test the first hypothesis, that connectivity is positively associated with species occurrence, we used Bayesian hierarchical models with the *brms* R package (Bürkner 2017). We used the species‐level connectivity effects (i.e., connectivity coefficients from the *inlabru* models) and fitted intercept‐only, random‐effects regression models, including the standard deviation of the fixed effect as measurement error in the response to account for model uncertainty. We included both recording scheme and individual species as random effects. See Supporting Information: Methods D for model parameterisation.

To explore the interplay between cover and connectivity effects, we also fitted models estimating broadleaf cover and broadleaf cover:connectivity interaction effects. We then used the estimated pooled effects across all species for broadleaf cover, connectivity and broadleaf cover:connectivity interactions to predict estimated species occurrence across broadleaf cover–connectivity space.

In addition to the main analysis, we undertook three additional analyses to explore the robustness of our findings in more depth. We know that cover and connectivity are linked and so, to separate out their effects on species, woodland landscapes were classified into four cover quartiles, excluding landscapes without any broadleaf cover (first quartile: 0.01%–1.75% broadleaf cover; second quartile: 1.75%–5.18% broadleaf cover, third quartile: 5.18%–13.25% broadleaf cover, fourth quartile: 13.25%–100% broadleaf cover); Figure S9. The main analysis was repeated for each quartile but with all cover and cover–connectivity interaction terms removed from the model. We spatially subset the entire dataset to only 1 × 1 km landscapes within the quartile such that 1 × 1 km cells outside these areas were only considered for the mesh fitting process for the spatial field.

Our analyses assume species have linear relationships with woodland availability but we tested for potential non‐linear associations with cover by repeating the main analysis and including a broadleaf cover quadratic term. We further tested for any effects of non‐linear relationships in our results by repeating the main analysis but spatially subsetting all data to include only 1 × 1 km landscapes under 30% cover. We did this because previous work has identified that most species have linearly positive associations with woodland cover below 30% cover (Bowler et al. 2023).

## 3 Results

### 3.1 Species Are Positively Associated With Woodland Connectivity

**FIGURE 1:**
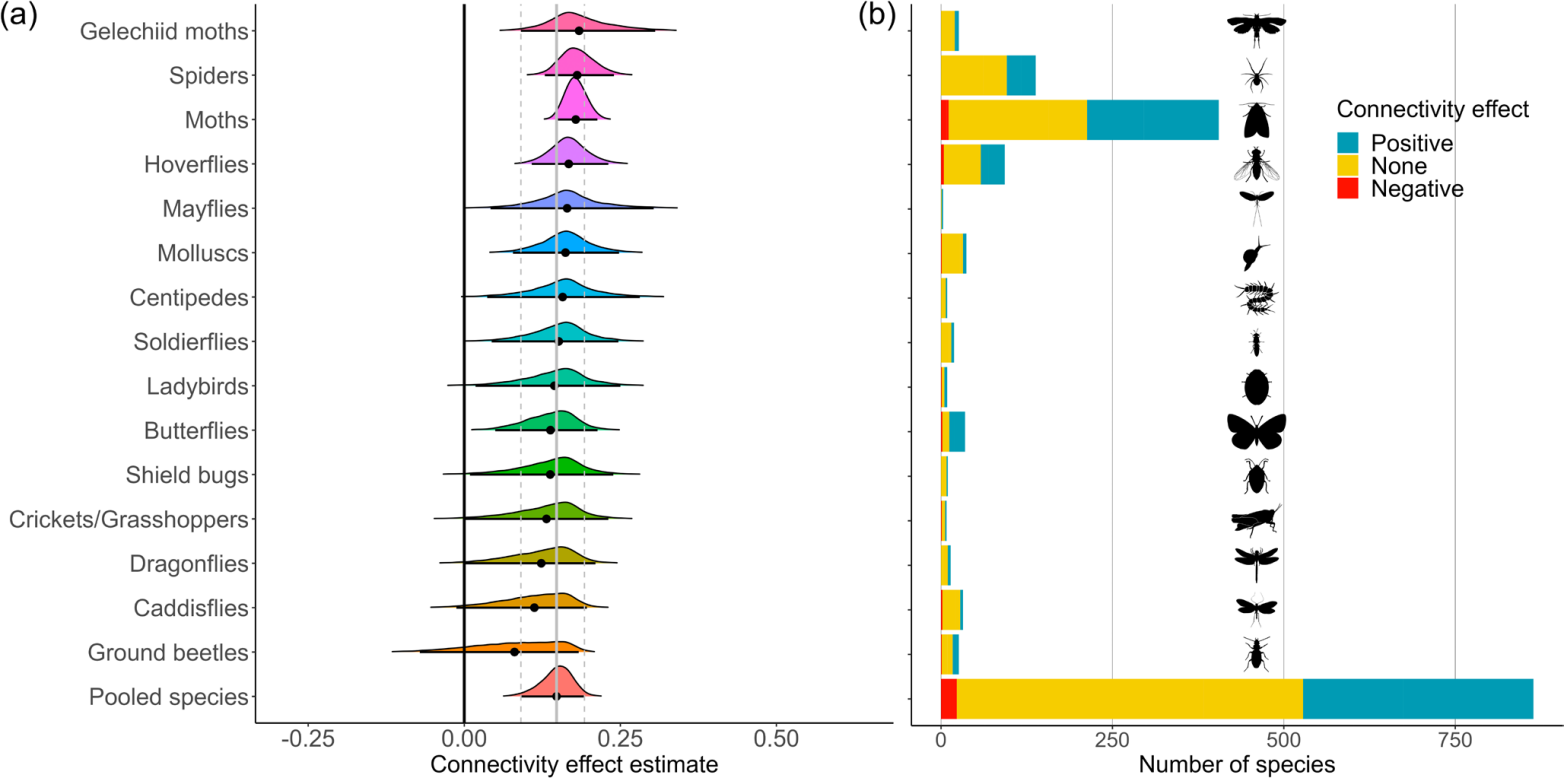
Connectivity effect sizes for broadleaf‐associated invertebrate species, after accounting for woodland cover (connectivity effects occur in the presence of interactions with broadleaf and coniferous cover, so the credible interval is valid at the mean cover values). (a) Estimated effect sizes based on Bayesian meta‐analysis of models showed a range of values. Density plots sorted by point estimates (black circles) with credible intervals (horizontal black lines) shown, and the solid and dotted grey lines indicating the estimated pooled estimate and credible interval, respectively. Spatial variables were standardised, hence effect size shown when broadleaf cover is at the mean value of 5.54%. (b) Individual species effect sizes were more often positive (credible interval entirely above zero) than negative (credible interval entirely below zero) for different recording schemes.

Of the 864 broadleaf‐associated species, from 15 invertebrate recording schemes, 336 species (38.9%) showed a positive effect of connectivity (95% credible interval entirely above zero), 23 species (2.7%) showed a negative effect (95% credible interval entirely below zero), and 505 (58.4%) were neither positive nor negative (Figure 1b). Overall, the meta‐analysis revealed that these broadleaf‐associated species are, on average, strongly affected by woodland cover as expected (Figure S11), but with an additional effect of connectivity such that broadleaf woodland‐associated species are more likely to occur in landscapes with better‐connected woodland (Table 1; Figure 1a). This positive effect of connectivity occurs in the presence of interactions with broadleaf and coniferous cover (Figure S10), so the credible interval is valid at the mean cover values.

**TABLE 1:** Pooled species effects from meta‐analysis of the individual species results for broadleaf‐associated species. See Figure 1 and Figures S11 and S12 for the results for the individual recording schemes.

	Estimate of pooled effect	95% CI
Connectivity	0.148	0.091, 0.192
Broadleaf cover	0.246	0.198, 0.292
Broadleaf cover:connectivity	−0.050	−0.60, −0.037

Between‐species heterogeneity (= 0.33 [0.31–0.35]) in the connectivity effect was much greater than between‐recording scheme heterogeneity (= 0.03 [0.00–0.13]). Despite considerable variation among species there were still differences between recording schemes, varying from a slightly positive estimated effect of connectivity for ground beetles (0.081, 95% Credible Interval: −0.071, 0.183) to a strongly positive effect for gelechiid moths (0.184, CI: 0.091, 0.306; Figure 1a).

When the analysis was restricted to landscapes with broadleaf cover under 30%, below which we expect linear cover associations, the results were similar [pooled effect estimates: connectivity, 0.220 (0.152, 0.290), Figure S14; cover, 0.258 (0.203, 0.309); cover:connectivity, −0.09 (−0.115, −0.069)]. The findings were also broadly similar when we included a quadratic broadleaf cover term to account for non‐linear associations [pooled effect estimates: connectivity, 0.052 (0.003, 0.107), Figure S15; cover, 0.342 (0.265, 0.415); cover quadratic, −0.026 (−0.034, −0.012); cover:connectivity, −0.014 (−0.247, −0.005)]. The pooled effect credible interval estimate for connectivity was positive, although this was lower than for the main analyses assuming linear associations with cover, and the ranking of recording schemes in terms of the strength of the connectivity effect differed.

### 3.2 Interactions Between Woodland Connectivity and Cover

**FIGURE 2:**
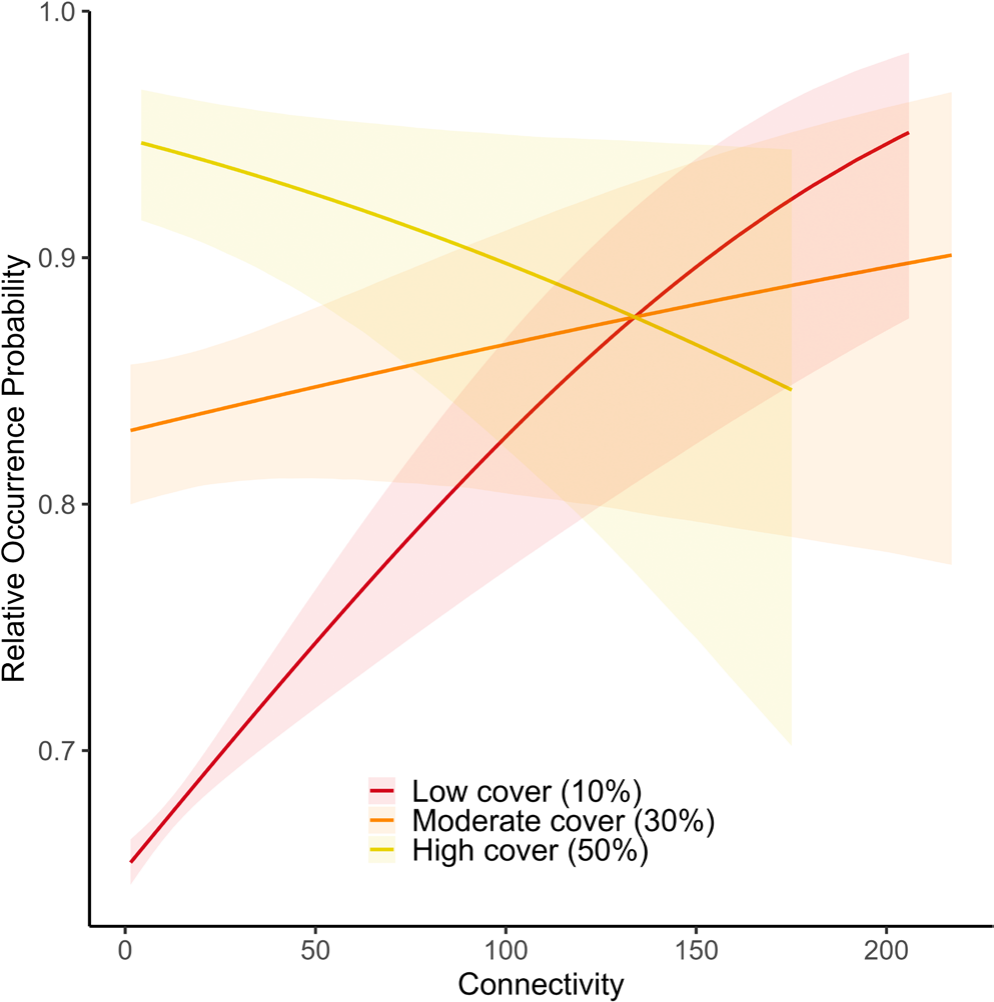
Interaction between broadleaf woodland cover and woodland connectivity (mean cumulative current flow) using estimated effect sizes from Bayesian meta‐analysis. Occurrence probability estimates were calculated by taking the effect size estimate draws from the separate connectivity, broadleaf cover, and broadleaf cover:connectivity meta‐analysis models, and using these to generate a prediction for each cover/connectivity combination. Darker lines show mean prediction and fainter ribbons show the 95% credible interval. Lines beyond the limits of observed values within UK landscapes for each cover value are not shown.

Broadleaf cover and woodland connectivity interacted negatively (Table 1) and this was consistent across recording schemes (Figures S10 and S12). In total, 62.3% of broadleaf‐associated species had a negative interaction (CI < 0), with 2.0% positive (CI > 0), and 35.8% with the CI crossing 0. The pooled negative interaction effect estimate meant that increasing woodland connectivity has the greatest positive benefits for broadleaf‐associated species when broadleaf cover is low (Figure S13). For example, if a landscape has broadleaf woodland cover of 10%, then increased connectivity is predicted to have a large positive effect, but this lessens as cover increases to 30%, and at 50% (exceptionally high woodland cover in the UK) the effect is negative, albeit with large uncertainty (Figure 2).

When the analysis was repeated separately for each of the four broadleaf cover quartiles, the results for each quartile were similar (pooled effect connectivity estimates: 0.01%–1.75% cover, 0.0873 (0.016, 0.146), Figure S16; 1.75%–5.18% cover, 0.102 (0.028, 0.165), Figure S17; 5.18%–13.25% cover, 0.034 (−0.037, 0.096), Figure S18; 13.25%–100% cover, 0.055 (0.038, 0.069), Figure S19). This indicates conclusions about the benefits of connectivity are evident regardless of the amount of woodland present, although given the large interval of the highest quartile due to the distribution of broadleaf cover in the UK (Figure S9), it is likely this quartile captured cover as well as connectivity effects (Figure 2). Interaction between broadleaf woodland cover and woodland connectivity (mean cumulative current flow) using estimated effect sizes from Bayesian meta‐analysis. Occurrence probability estimates were calculated by taking the effect size estimate draws from the separate connectivity, broadleaf cover and broadleaf cover: connectivity meta‐analysis models, and using these to generate a prediction for each cover/connectivity combination. Darker lines show mean prediction and fainter ribbons show the 95% credible interval. Lines beyond the limits of observed values within UK landscapes for each cover value are not shown.

## 4 Discussion

We quantified the importance of woodland connectivity for a wide range of invertebrate species in the UK. We were able to make use of high‐quality datasets of occurrences of 3277 invertebrates species collected by thousands of expert volunteers over the past 20+ years, demonstrating the value of unstructured ‘citizen science’ recording for detailed analysis of the current status of species, and their spatial distributions. We modelled the importance of woodland connectivity for the occurrence of broadleaf‐associated species and found that the pooled effects of woodland connectivity were positive overall, although these varied greatly among our 15 invertebrate recording schemes. Hence we conclude there are potential benefits to be gained for woodland biodiversity from improving connectivity, above the greater effects of increasing woodland cover. A significant interaction term between broadleaf cover and connectivity revealed that connectivity benefits were greatest for species occurrence in landscapes with low broadleaf cover levels which constitutes most of the UK.

We found that moths, spiders, and hoverflies benefited most from woodland connectivity. Conversely, the three groups in our study that benefited least from connectivity, that is, lowest estimated effect sizes, were ground beetles, dragonflies and caddisflies (Figure 1a). The latter two have aquatic larval life stages and so are influenced more by suitable aquatic habitats than by the connectivity of woodland habitats used relatively briefly by the adults. However, despite overall positive connectivity benefits for all recording schemes, species responses varied greatly within recording schemes, and nearly all recording schemes contained some species with a negative association with connectivity.

The large range of effect sizes we observed among taxa is presumably due to variation in multiple interacting traits at the species level that affect responses to connectivity, such as dispersal ability and trophic level (Gordon et al. 2023; Wang et al. 2024) but see Martin et al. (2023). This large amount of variation we observe with taxa highlights that generalising single species connectivity models to other species should be interpreted cautiously (Liczner et al. 2024), that is, connectivity benefits for one species may not be seen for another, and that any surrogates should not necessarily be chosen based on taxonomy.

Connectivity for broadleaf species was found to have the overall largest effects when there are low levels of woodland cover within the landscape. Species may be more sensitive to connectivity in low cover landscapes as metapopulation dynamics become more important as local extinction becomes more likely. Connectivity is then able to ameliorate potential extinctions by increasing dispersal between habitat fragments in the landscape, or leading to larger habitat fragments that are more resilient to extinction events (Hanski 1998; Herrault et al. 2016; Uroy et al. 2023). Alternatively, given the low woodland cover in the majority of UK landscapes, better‐connected landscapes may also be associated with other beneficial landscape features affecting species, such as co‐occurring with more heterogeneous landscapes for multiple habitat types (Clauzel et al. 2024; Hackett et al. 2024; Maskell et al. 2019). Better‐connected landscapes may reduce edge effects, which alter the microclimate conditions and vegetation structure at woodland boundaries, affecting the woodland species that can occur there (Dennis, Huck, Holt, Bispo, et al. 2024; Dennis, Huck, Holt and McHenry 2024). The mechanisms underpinning positive effects could be species‐specific and would make a good subject for future research.

Our main results show it is important to consider landscape spatial configuration alongside woodland cover in predicting the occurrence of broadleaf‐associated species. In landscapes with low woodland cover and connectivity, such as in the UK, our findings suggest that maximally increasing connectivity when undertaking woodland creation (i.e., considering the spatial configuration of schemes to increase woodland cover locally) will have additional benefits for many invertebrates. We find benefits of connectivity are primarily seen at low levels of broadleaf cover, which suggests connectivity may be moderating the negative effects of fragmentation when the amount of woodland cover in the landscape is low, supporting limited existing empirical evidence that this occurs below 20%–30% (Fahrig 2017). However, species which prefer high woodland cover landscapes may have been historically filtered out in the UK (Betts et al. 2019), and benefits of connectivity may be evident at higher levels of woodland cover in other countries.

Here we look at biodiversity benefits solely in the context of species occurrence, but connectivity has other benefits too, such as providing opportunities for range shifting by species in response to climate change (Hill et al. 2001; Hodgson et al. 2022). We also studied connectivity benefits for focal habitat‐associated species (broadleaf woodland), but benefits may differ for subsets of species, for example, rare species of conservation concern (Bowler et al. 2023), or between specialist and generalist species (Chetcuti et al. 2020). Although we find an overall positive effect of connectivity, a few broadleaf‐associated species (23; 2.7%) had significantly negative associations with connectivity, and there are other potential negative impacts of connectivity, such as increased risk of spread of pests and disease (Maguire et al. 2015). It is possible that increasing connectivity for one habitat can also reduce connectivity of other habitats, potentially benefiting focal habitat species at the cost of others. For example, open‐associated species might be expected to decrease in occurrence as a result of increased woodland connectivity, although we did not find this at the scale of our analysis (Figure S8).

In our study, we have considered woodland to be homogeneous in terms of quality. However, there will be woodland features such as age, structure, tree species composition, canopy density, woodland rides, and type of management and condition that affect species occurrences (Blumgart et al. 2022; Fuentes‐Montemayor et al. 2022; Spitzer et al. 2008; Staab et al. 2023). Features outside woodland could also affect connectivity, such as ancient trees outside woodlands, hedgerows and wood‐pasture (Liu et al. 2023; Nolan et al. 2021; Tiang et al. 2021), that may be important population sources of woodland species, as well as aids to dispersal. Given the UK‐wide extent of our study, it was not possible to include this additional information on woodland features, but this may be interesting to include in analyses at regional scales where these data are available, and providing more information on the circumstances when connectivity is most beneficial.

In summary, there was substantial variation among species but, overall, woodland connectivity was positively associated with broadleaf‐associated invertebrate species occurrence in the UK. This was evident even after taking the larger effect of woodland cover into account. In the UK, we found that woodland connectivity benefits were greatest in landscapes with low woodland cover. This suggests that benefits for biodiversity can be maximised by designing woodland creation schemes to increase connectivity in regions with low woodland cover.

## Author Contributions

C.A.C., C.M.B., D.E.B., M.J.O.P., J.K.H., P.C.L.W. designed the study. R.H. processed the species presence records. C.A.C., C.M.B., D.E.B., M.J.O.P. conducted the analysis. C.A.C. and D.E.B. wrote the first draft of the manuscript. All authors contributed substantially to discussions and to revising the paper.

### Peer Review

The peer review history for this article is available at https://www.webofscience.com/api/gateway/wos/peer‐review/10.1111/ele.70131.

## Supporting information


Data S1.

## Data Availability

The code and data that support the findings of this study are openly available in figshare at https://figshare.com/s/87fe97433cded40afe47. All covariate data used in the analysis are publicly available: climate data from https://data.ceda.ac.uk/badc/ukmo‐hadobs/data/insitu/MOHC/HadOBS/HadUK‐Grid/v1.1.0.0, soil moisture data from https://catalogue.ceh.ac.uk/documents/c9a85f7c‐45e2‐4201‐af82‐4c833b3f2c5f, landcover data from https://www.ceh.ac.uk/data/ukceh‐land‐cover‐maps. We provide code to process these open datasets in the figshare repository as well as the processed versions of these data. Raw species data is available upon request from Biological Records Centre (BRC: https://www.brc.ac.uk/) and Centre for Environmental Data and Recording (CeDAR: https://www.nationalmuseumsni.org/cedar) under licence from the data owners, as our licence to use these prohibits direct sharing of the raw data, though the authors can provide the archived dataset to colleagues seeking to replicate this process who have approved requests from the BRC and CeDAR. All code required to run the processing and distribution modelling of species data are provided in the figshare repository, where we also provide processed covariate datasets and a single anonymised species dataset to enable replication of the species occurrence modelling. All data required for the meta‐analyses is provided.
