# Supplementary material for: Connectivity Benefits Most Woodland Invertebrate Species but Only in Landscapes With Low Woodland Cover
Source: Ecol Lett. 2025 May 19;28(5):e70131. doi: 10.1111/ele.70131 (PMC12087529; doi:10.1111/ele.70131)
Supplement: Supplementary file 1 — Data S1. [file ELE-28-0-s001.docx]

**SUPPLEMENTARY INFORMATION**

**Supplementary Methods**

1. ***Analysing species occurrence data***

Recording schemes were contacted to request permission for their data to be used, where a scheme dataset was already available (such as through the NBN Atlas), and asked if they would like to provide an updated version for this project. We also contacted schemes without publicly available datasets to request a data export for this project. Data was provided either directly from the scheme, or we had permission for reuse from a previous study, or it was available through NBN and Indicia.

1. ***Quantifying connectivity***

 As part of a circuit-models, current is injected into all source pixels (i.e. all woodland pixels) within a set radius of the focal pixel, and flows to the ground pixel (i.e. the focal woodland pixel) across the landscape (a mix of woodland and non-woodland cells). For each focal woodland pixel, the total source strength was set at 1 which was evenly distributed to all source pixels within the radius. For each terrestrial non-woodland pixel, this was set to 0, i.e. omniscape was not run for them but they can still have positive connectivity values generated from other omniscape calculations within the radius. Any non-terrestrial cells (e.g. marine) were masked out of the analysis.

The cumulative current flow across all iterations of the moving window provides an estimate of the movement intensity of organisms through the landscape. Hence the moving window radius and resistance surfaces are key parameters that set the maximum distance between source and target (ground) pixels and non-habitat resistance respectively. See Landau et al. (2021) for full description of methods.

To explore the consequences of different buffer distances and resistance values in addition to the chosen buffer distance of 4km (block size = 15), we also calculated connectivity using buffer distances of 500m (block size = 1), 1km (block size = 3) and 2km (block size = 5). Block size refers to the aggregation factor applied to the source raster to significantly reduce compute times with negligible differences in output. In addition to the chosen resistance value of 100 for non-woodland pixels, we also calculated connectivity using resistance values of 10 and 1000. These were highly correlated with the selected connectivity parameters (Fig. S2).

1. ***Exploring species associations with connectivity***

$$y_{i,t,j}=Bernoulli(\psi_{i,t, j})$$

Eq. 1

$$cloglog\left( \psi_{i,t, j} \right)=\beta_{0}+\beta_{1}{Connect}_{i,t}+\beta_{2}{CoverBL}_{i,t}+\beta_{3}{CoverCon}_{i,t}+\beta_{4}\left( {Connect}_{i,t}\times{CoverBL}_{i,t} \right)+\beta_{5}\left( {Connect}_{i,t}\times{CoverCon}_{i,t} \right)+\beta_{6}VLs{ingle}_{i,t,j}+\beta_{7}{VLshort}_{i,t,j}+u_{i,t,j}+\omega_{i,t}$$

Eq. 2

$$u_{i,t,j}={RW2(GDD5|\tau_{1})}_{i,t}+{RW2(WMIN|\tau_{2})}_{i,t}+{RW2(tasCV|\tau_{3})}_{i,t}+{RW2(RAIN|\tau_{4})}_{i,t}+{RW2(soilM|\tau_{5})}_{i,t}+cyclicRW2({week|\tau_{6})}_{j}$$

Eq.3

For each separately modelled species, the probability of occurrence was modelled using a Binomial distribution (Eq. 1) where $y_{i,t,j}$ represents the detections of the species within a 1km grid cell *i* in period *t*, on visit *j*.

Detection probability $\psi_{i,t,j}$ for each visit was modelled using a complementary log–log link function (cloglog) (Eq.2). Connectivity (*Connect*), broadleaf cover (*CoverBL*)and coniferous cover (*CoverCon*) were included as fixed effects, along with connectivity: broadleaf cover and connectivity: coniferous cover interactions. Survey effort was included as a fixed categorical variable with three levels (single, 1 species recorded (*VLsingle*); short, 2-3 species recorded (*VLshort*); or long, 4+ species recorded) with long as the reference level. A spatio-temporal field $\omega_{i,t}$was modelled as a spatial Gaussian random field with Matérn covariance function, and the two time periods were linked with an AR(1) model (see Morera-Pujol *et al.* (2022) for further details on the spatial field construction, and Wiethase *et al.* (2024) for further details on AR(1) model).

Random effects $u_{i,t,j}$ for climate variables were included as 2^nd^ order random walk random effects (RW2) (Gómez-Rubio 2020; Rue & Held 2005), as was week of year (cyclic) (Eq. 3). See Seaton *et al.* (2024) for an example of how random walk processes can be integrated into species distribution modelling. Exact specification of the RW2 model can be found here (<https://inla.r-inla-download.org/r-inla.org/doc/latent/rw2.pdf>). Penalised complexity priors were used for both the AR(1) and rw2 effects with parameters (σ^0^ = 0.5, α=0.01) where P(σ>σ^0^) = α.

1. **Testing the importance of connectivity using meta-analysis**

We set weakly informative priors such that pooled effect mean had distribution μ∼Normal(0,1) and between-study heterogeneity τ∼HalfCauchy(0,1), and 10000 iterations with 5000 warm up iterations (model parameters: adapt_delta = 0.999, stepsize = 0.001, max_treedepth = 20). Model convergence was checked using Rhat values, and for all models Rhat ≤ 1.00.


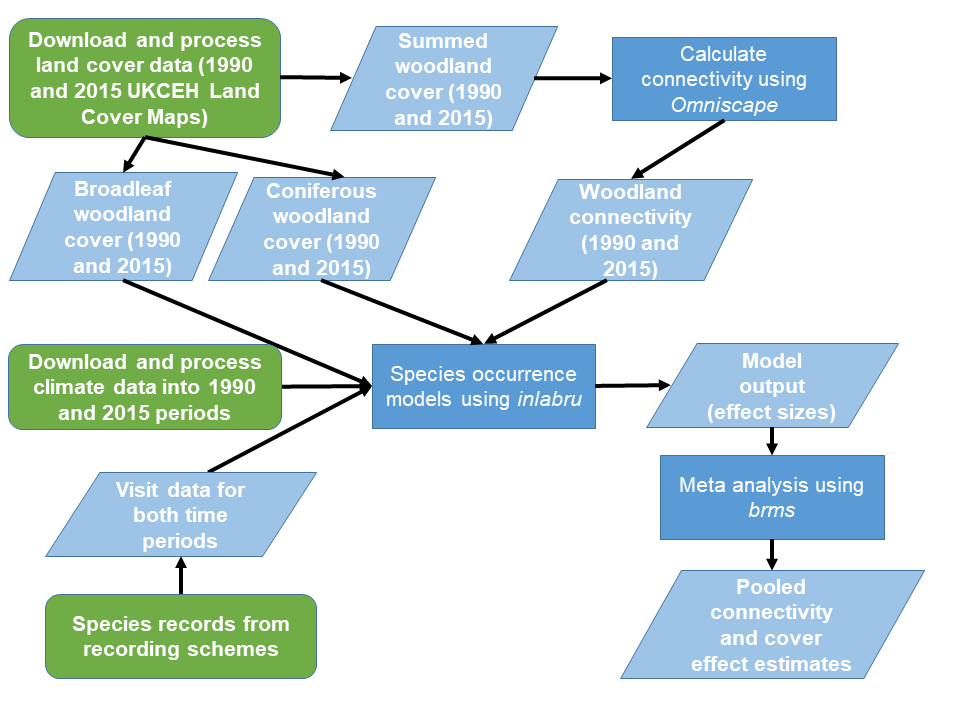


Figure S1. Outline of workflow used in the analysis.


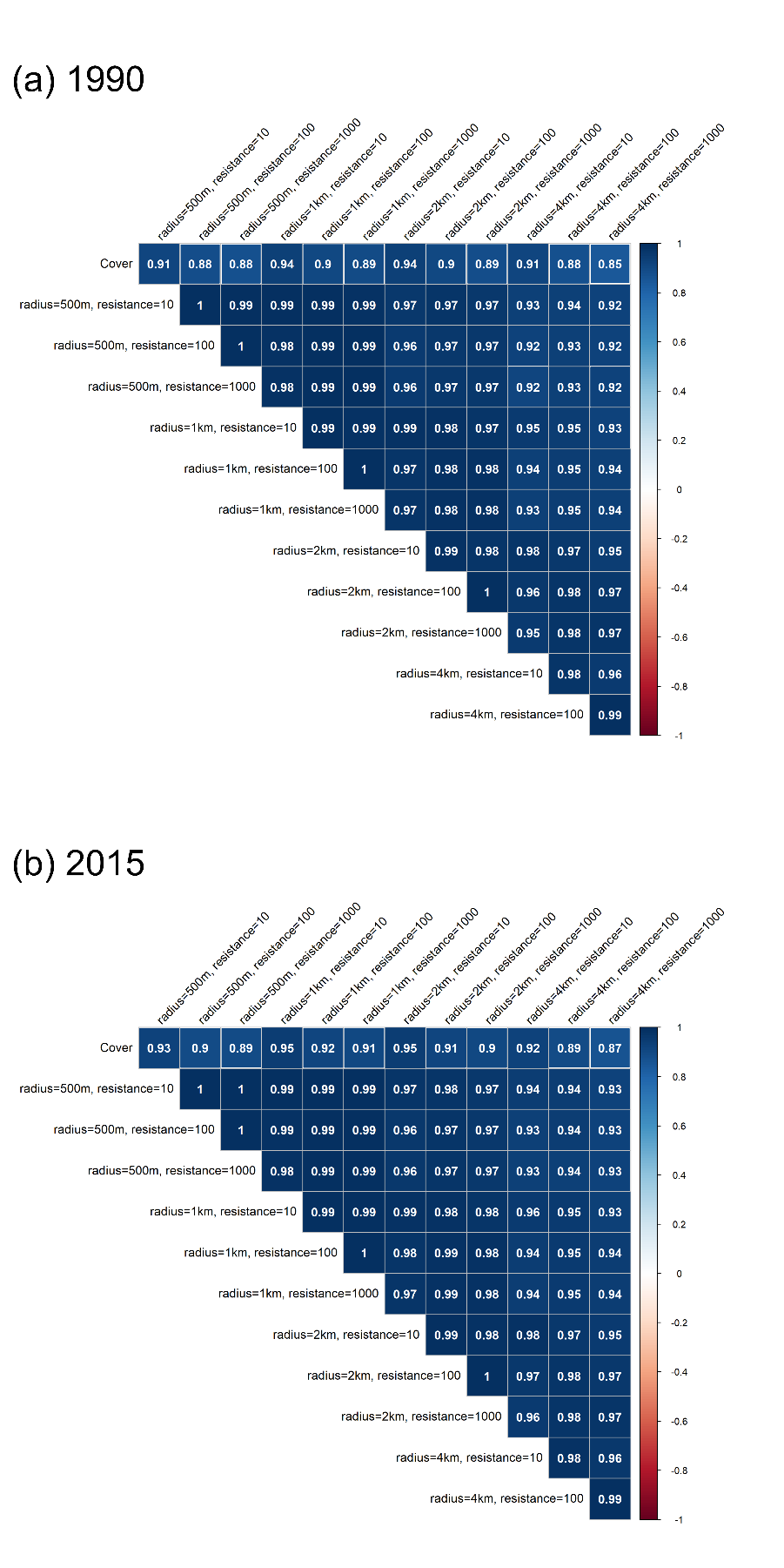


Figure S2. Correlation between woodland cover and different potential functional connectivity simulations generated by *omniscape* with different radius and resistance parameterisations for (a) 1990 and (b) 2015. Different parameterisations were highly correlated to the 4km radius, 100 resistance chosen for the analysis.


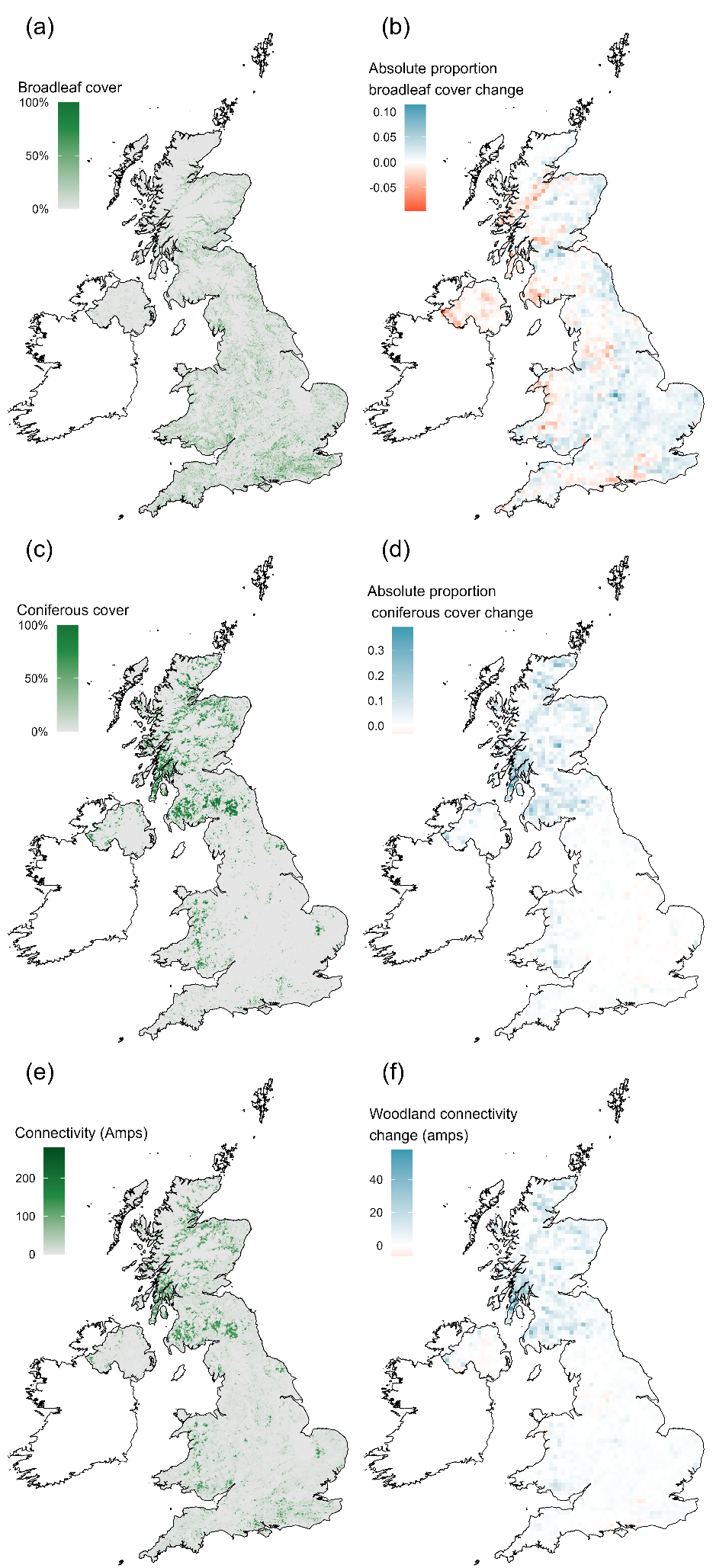


Figure S3. Broadleaf and coniferous woodland cover, and woodland connectivity. (a), (c) and (e) show proportion broadleaf cover, proportion coniferous cover, and woodland connectivity in 2015, respectively; and (b), (d) and (f) show absolute change from 1990-2015 in proportion broadleaf cover, proportion coniferous cover, and woodland connectivity, respectively. Broadleaf cover within the UK is generally low (<50%) and distributed throughout different regions, whereas coniferous cover has higher spatial heterogeneity with fewer, high coverage (>50%) patches concentrated in Wales and Scotland. Woodland connectivity reflected these spatial patterns with connectivity hotspots primarily in Scotland. Since 1990, small increases in broadleaf cover were seen across the UK with concentrations in the National Forest, in the Midlands, and in the central belt of Scotland, and small losses in Northern Ireland and west Scotland. Increases in coniferous cover since were greater than for broadleaf woodland, and were primarily concentrated in Scotland, as was connectivity change.


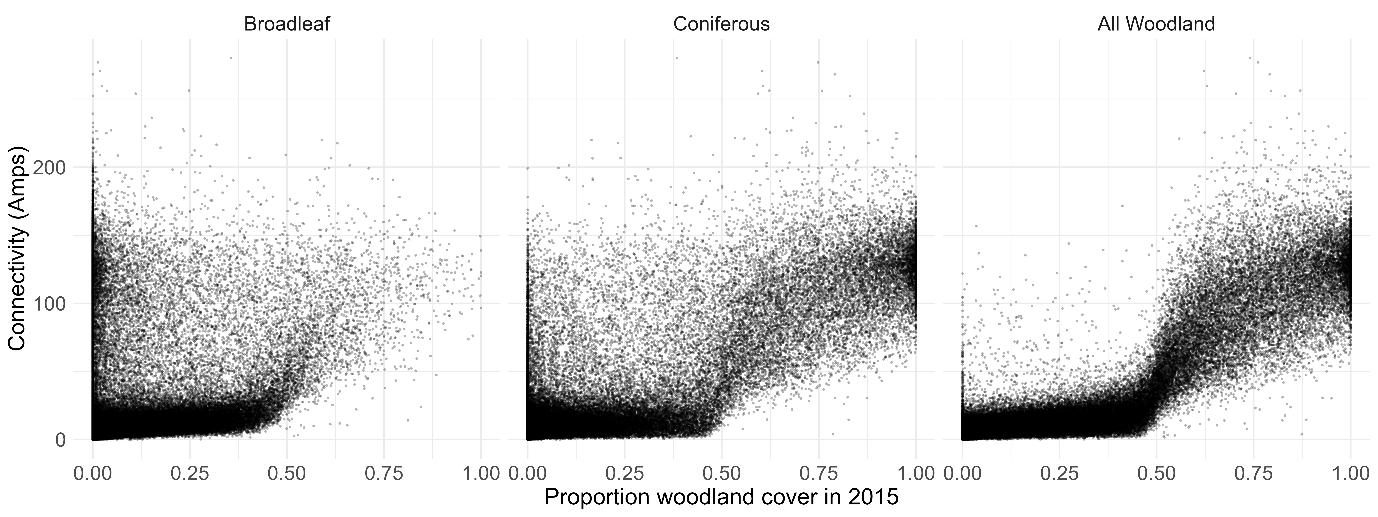


Figure S4. Relationship between woodland connectivity (all woodland combined, see Methods) and broadleaf, coniferous and total woodland cover in 2015. Each point represents a single 1x1km UK cell. Although there is a clear (non-linear) relationship between connectivity (all woodland) and cover (all woodland), this relationship is less strong when considering broadleaf woodland cover on its own.


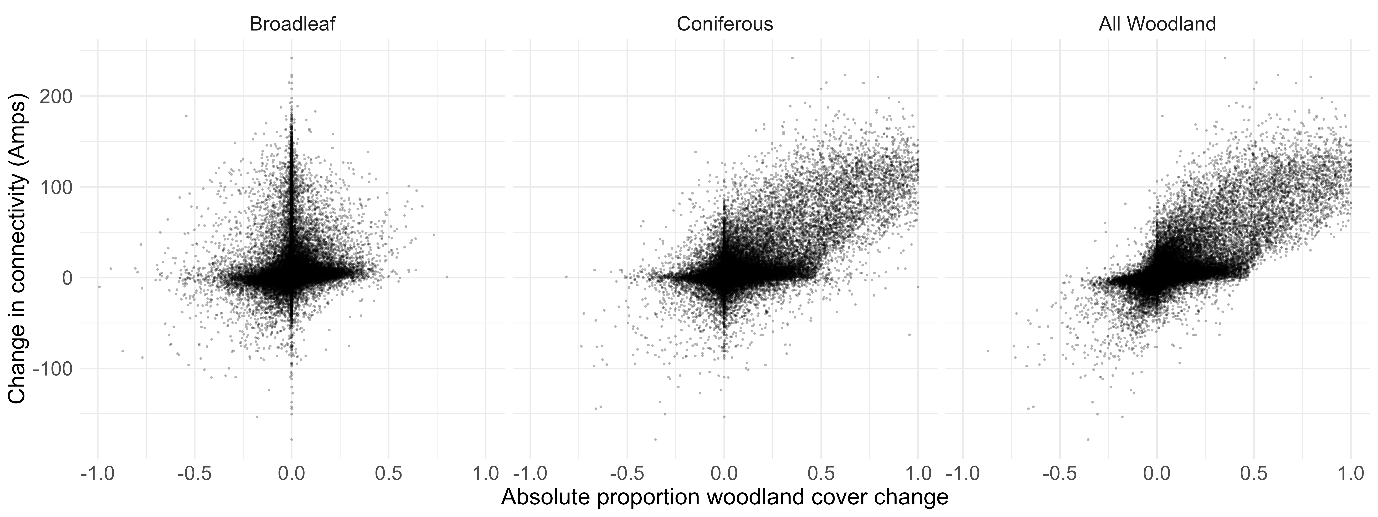


Figure S5. Relationship between change in woodland connectivity (all woodland combined, see Methods) between 1990 and 2015 and change in broadleaf, coniferous and total woodland cover in from 1990 to 2015. Each point represents a single 1x1km UK cell. Change in woodland cover since 1990 has primarily been driven increases in coniferous cover.

**
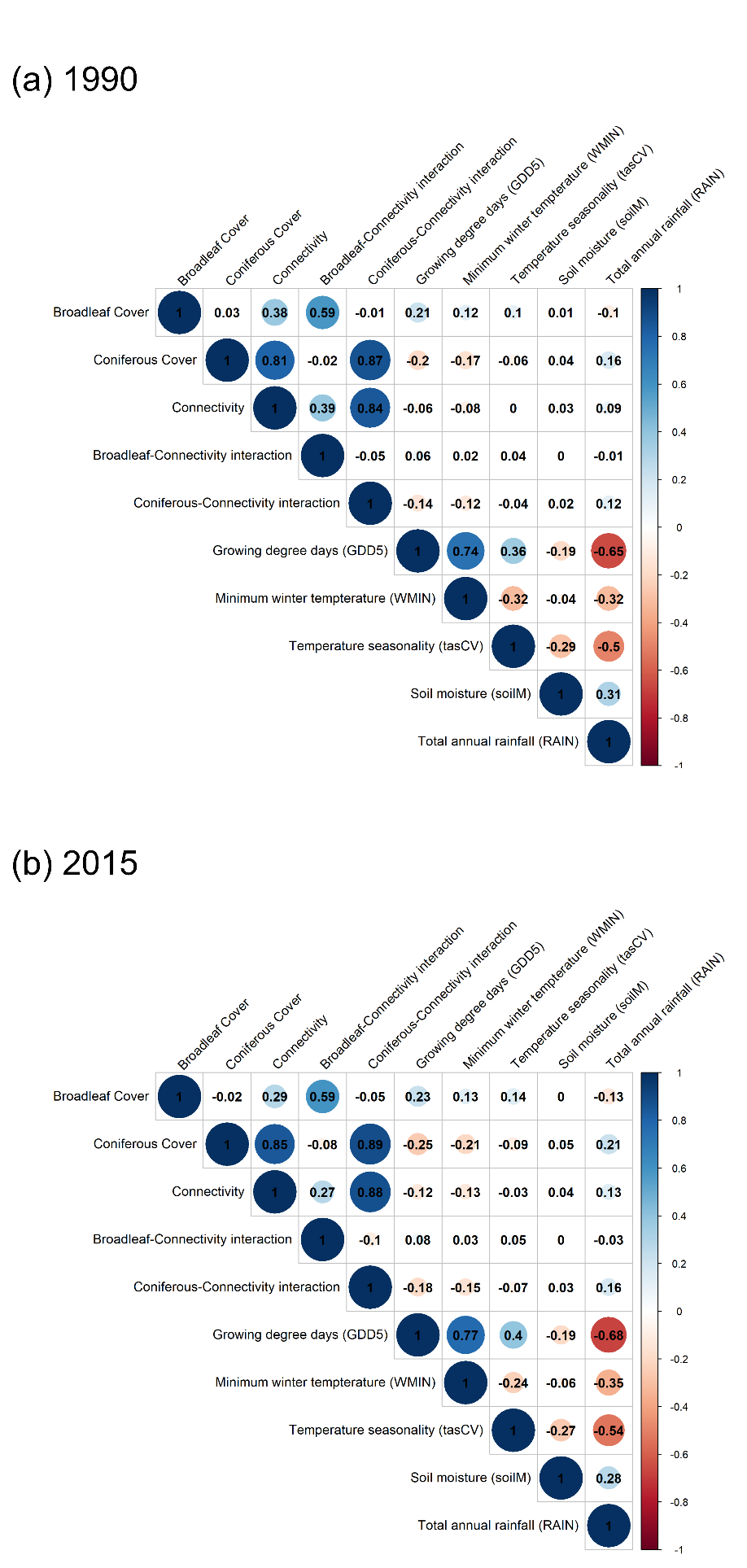
**

Figure S6. Correlation between cover, connectivity and climate variables included in species occurrence models for (a) 1990 and (b) 2015.


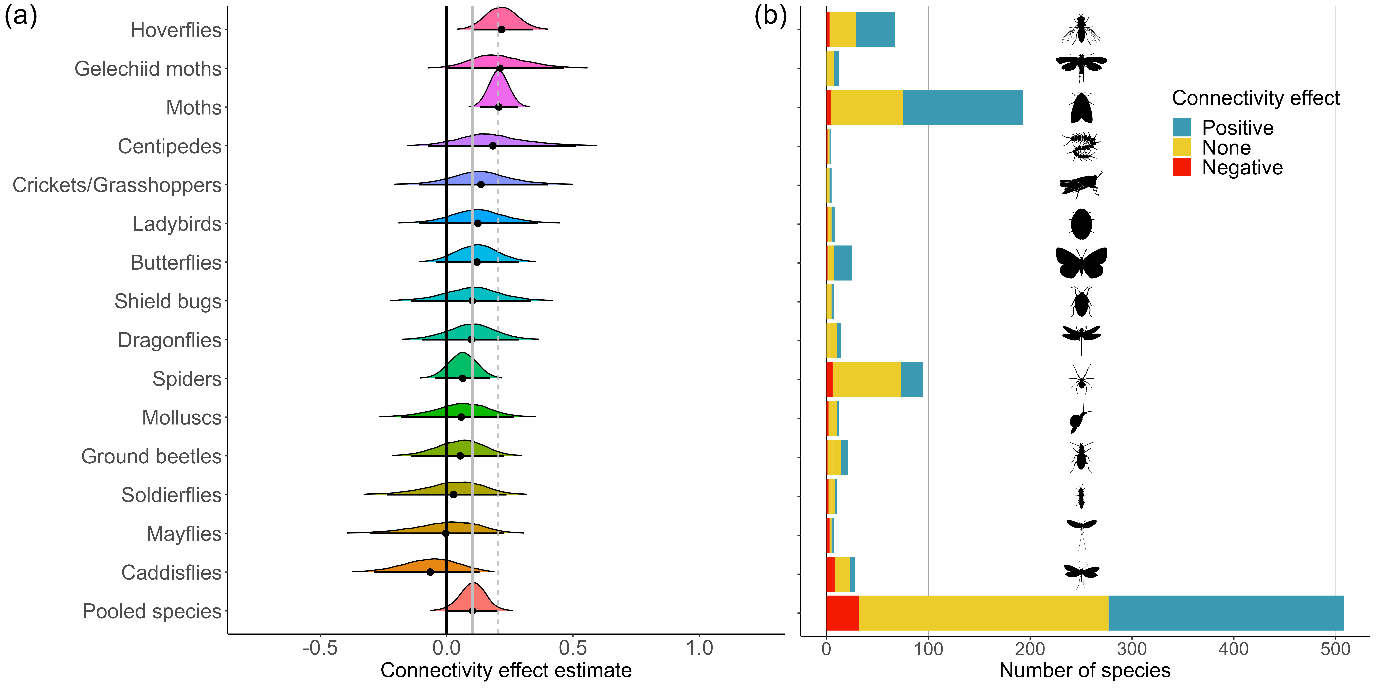
Figure S7. Connectivity effect sizes for coniferous-associated invertebrate species. (a) Density plots of estimated effect sizes sorted by point estimates (black circles) with credible intervals (horizontal black lines) shown, and the solid and dotted grey lines indicating the estimated pooled estimate and credible interval, respectively. (b) Individual species effect sizes for different recording schemes. We did not find the same positive associations with woodland connectivity for coniferous-associated species as we did with broadleaved species in the main analysis (the credible interval of the pooled species estimates for connectivity overlapped zero).


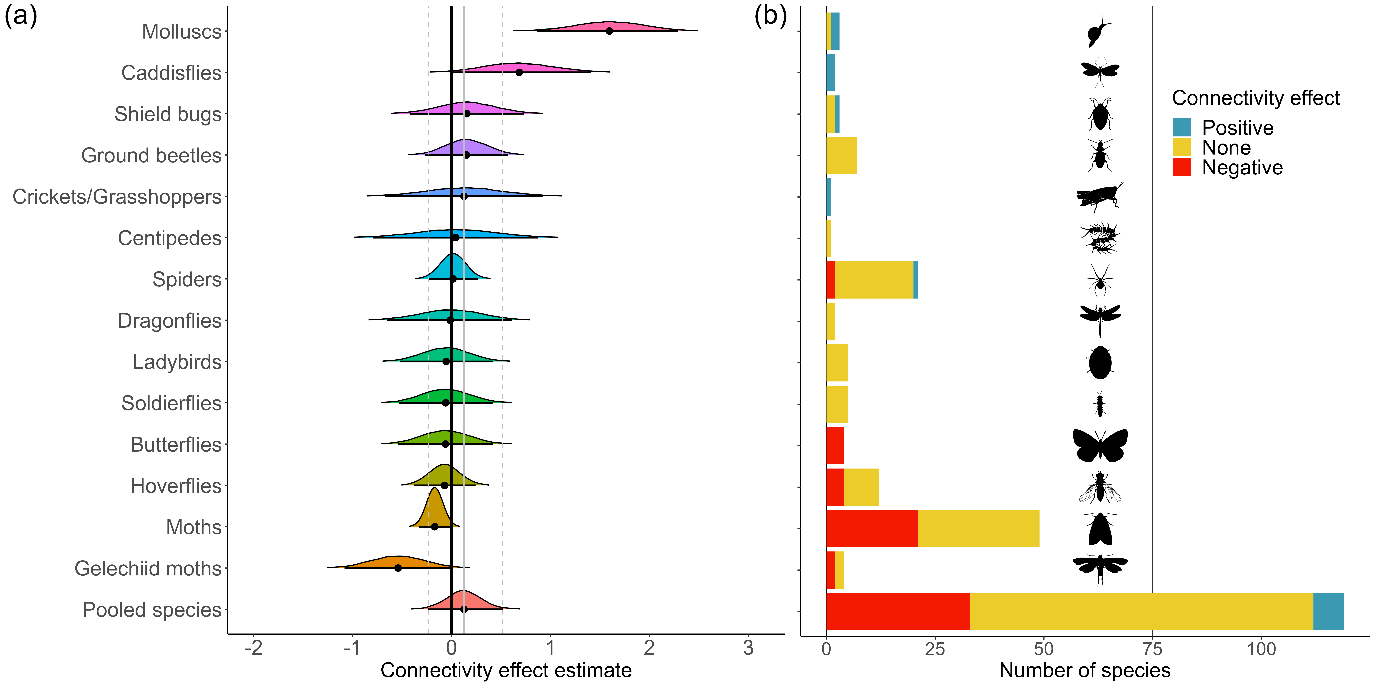


Figure S8. Connectivity effect sizes for woodland-avoiding invertebrate species. (a) Density plots of estimated effect sizes sorted by point estimates (black circles) with credible intervals (horizontal black lines) shown, and the solid and dotted grey lines indicating the estimated pooled estimate and credible interval, respectively. (b) Individual species effect sizes for different recording schemes. Unsurprisingly, woodland-avoiding species did not have a positive pooled estimate (the credible interval of the pooled species estimates for connectivity overlapped zero), but neither was it negative. Of the 15 recording schemes examined, moths and Gelechiid moths were the only groups that had a negative effect of connectivity with CI entirely below zero.


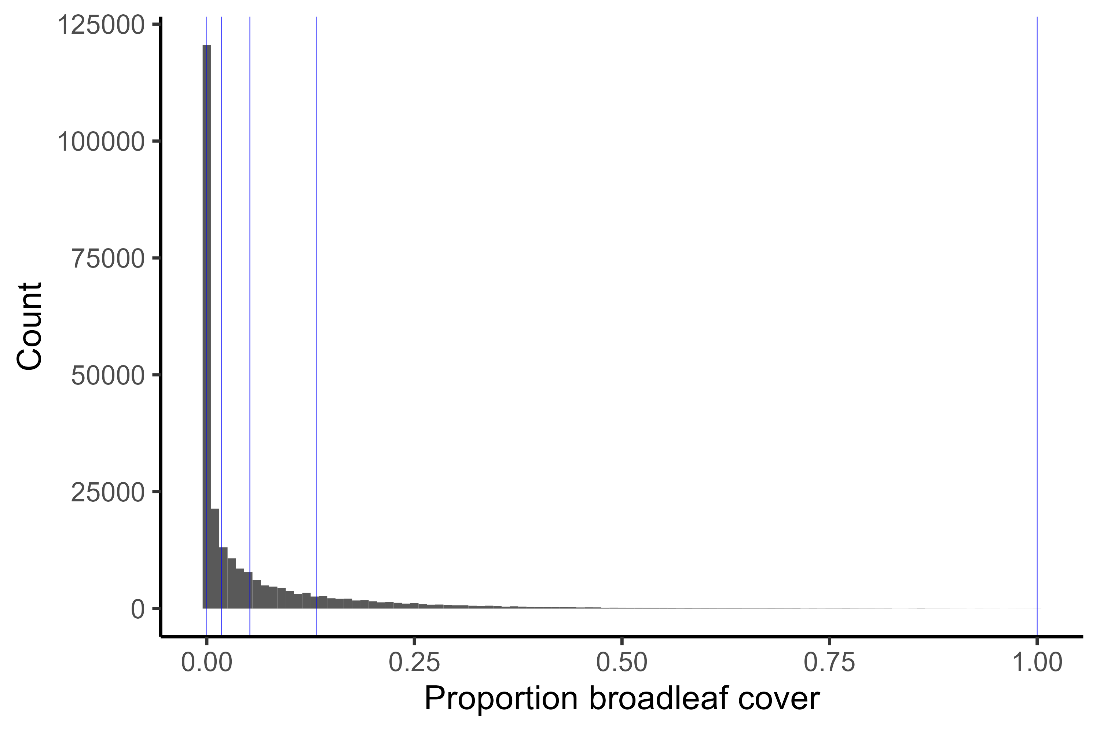


Figure S9. Broadleaf cover of each UK 1x1km cell. Blue vertical lines indicate quartiles of broadleaf woodland cover (excluding no cover values) used in the supplementary analysis. Landscapes in the UK typically have low levels of broadleaf cover.


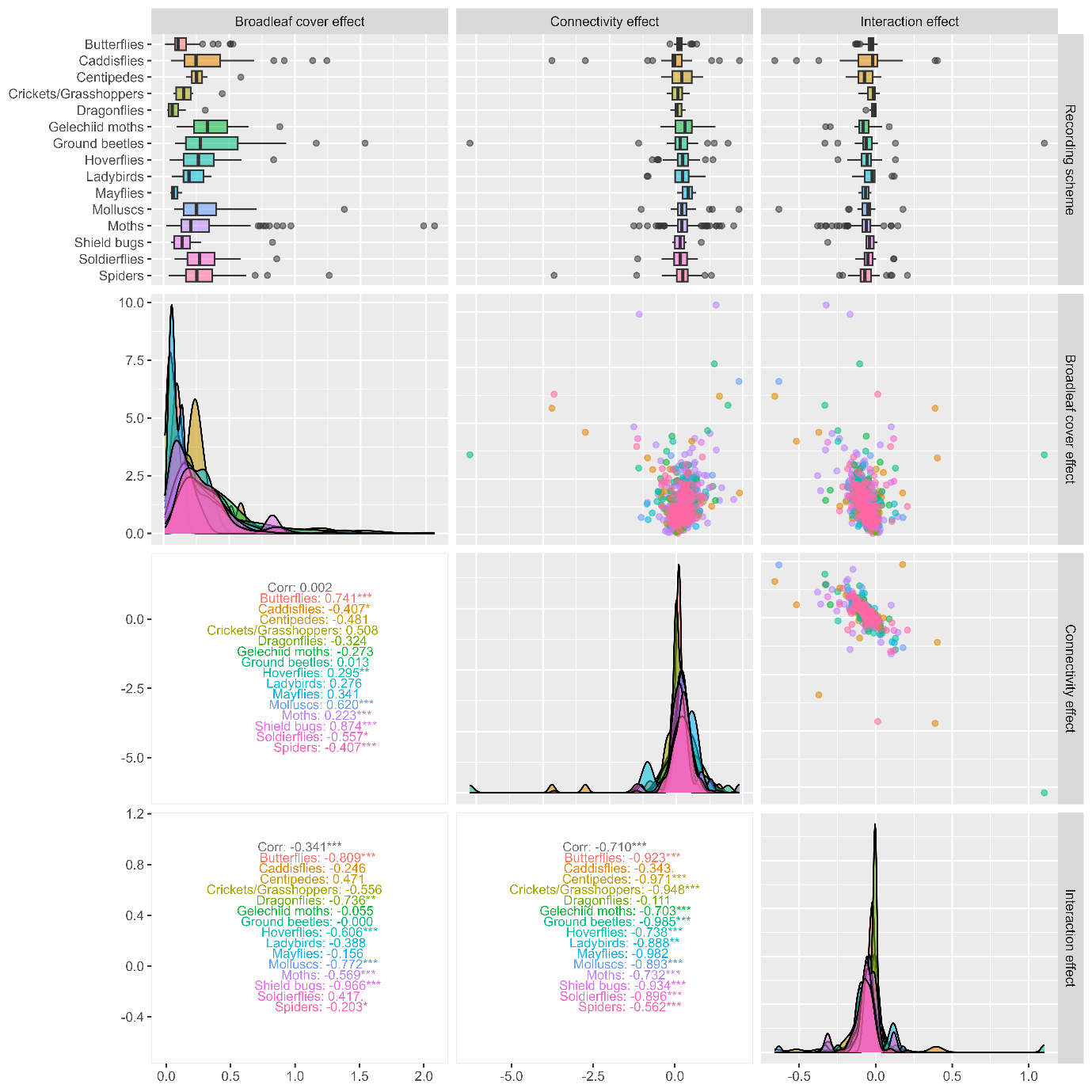


Figure S10. Raw effect sizes of broadleaf-associated species for broadleaf cover, connectivity, and broadleaf cover: connectivity interaction from individual species occurrence models. Broadleaf cover effects varied more than connectivity or cover:connectivity interaction effects, which were fairly consistent between recording schemes.


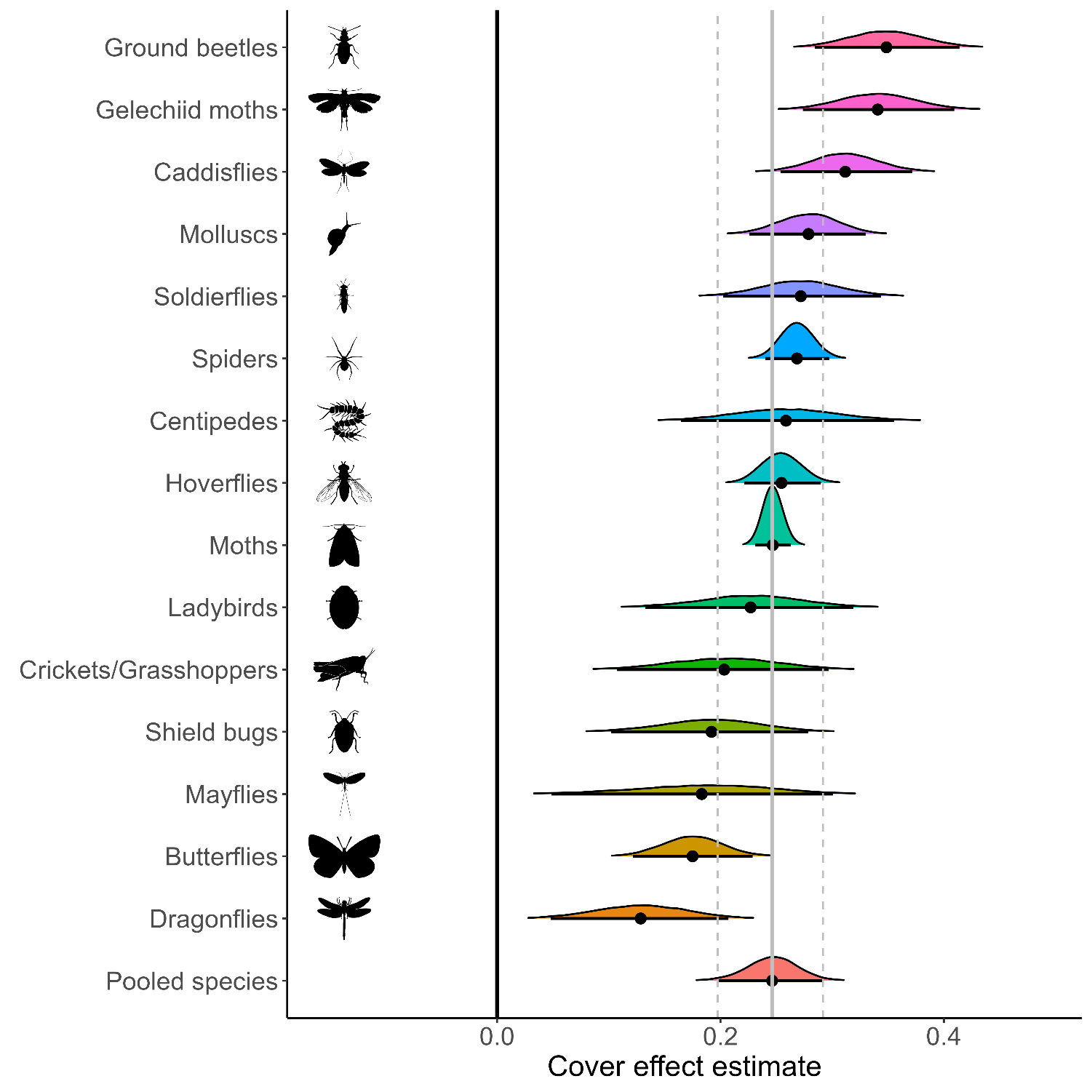


Figure S11. Estimated broadleaf-associated species broadleaf cover effect sizes based on Bayesian meta-analysis of models. Density plots sorted by point estimates (black circles) with credible intervals (horizontal black lines) shown, and the solid and dotted grey lines indicating the estimated pooled estimate and credible interval, respectively.


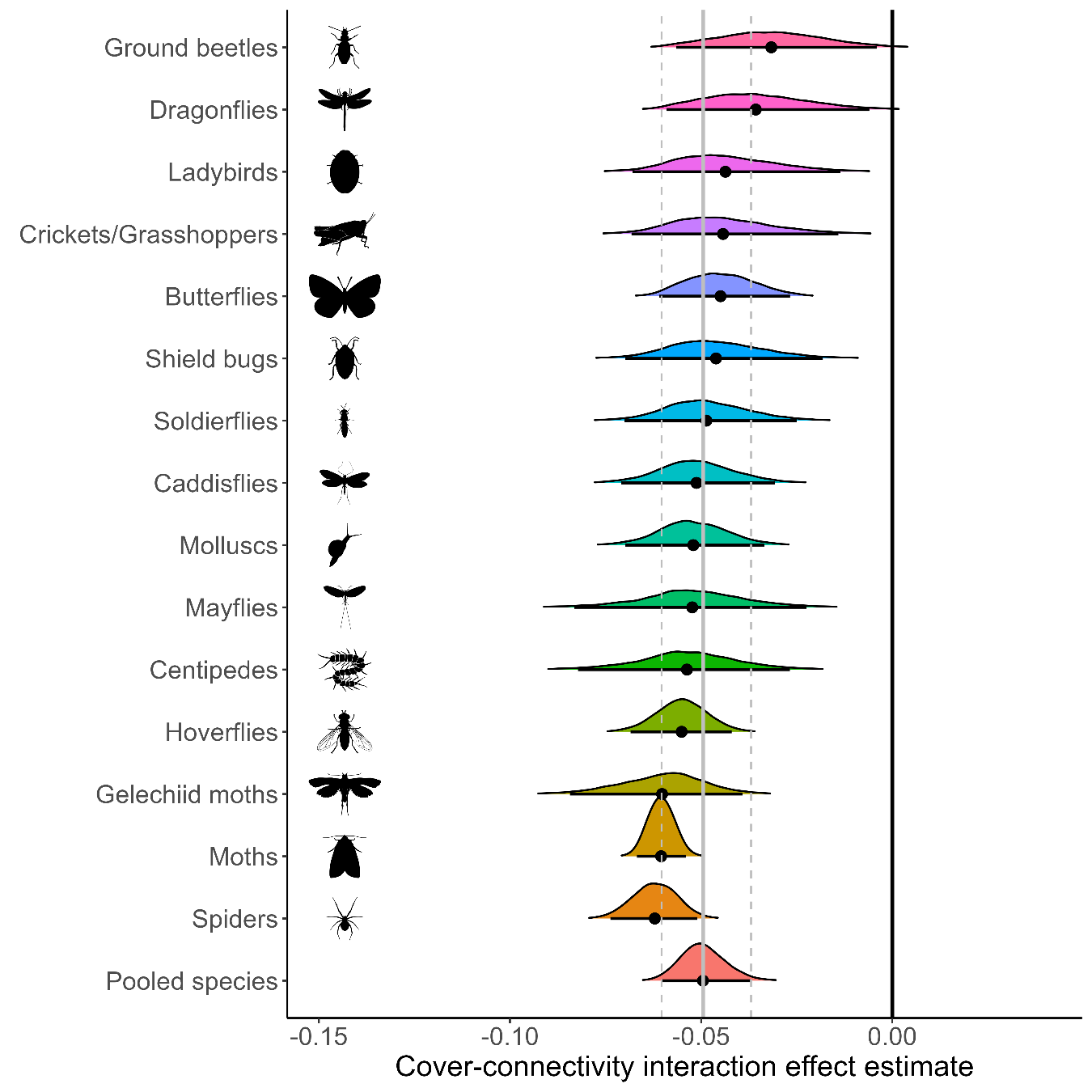


Figure S12. Estimated broadleaf-associated species broadleaf cover: connectivity interaction effect sizes based on Bayesian meta-analysis of models. Density plots sorted by point estimates (black circles) with credible intervals (horizontal black lines) shown, and the solid and dotted grey lines indicating the estimated pooled estimate and credible interval, respectively.


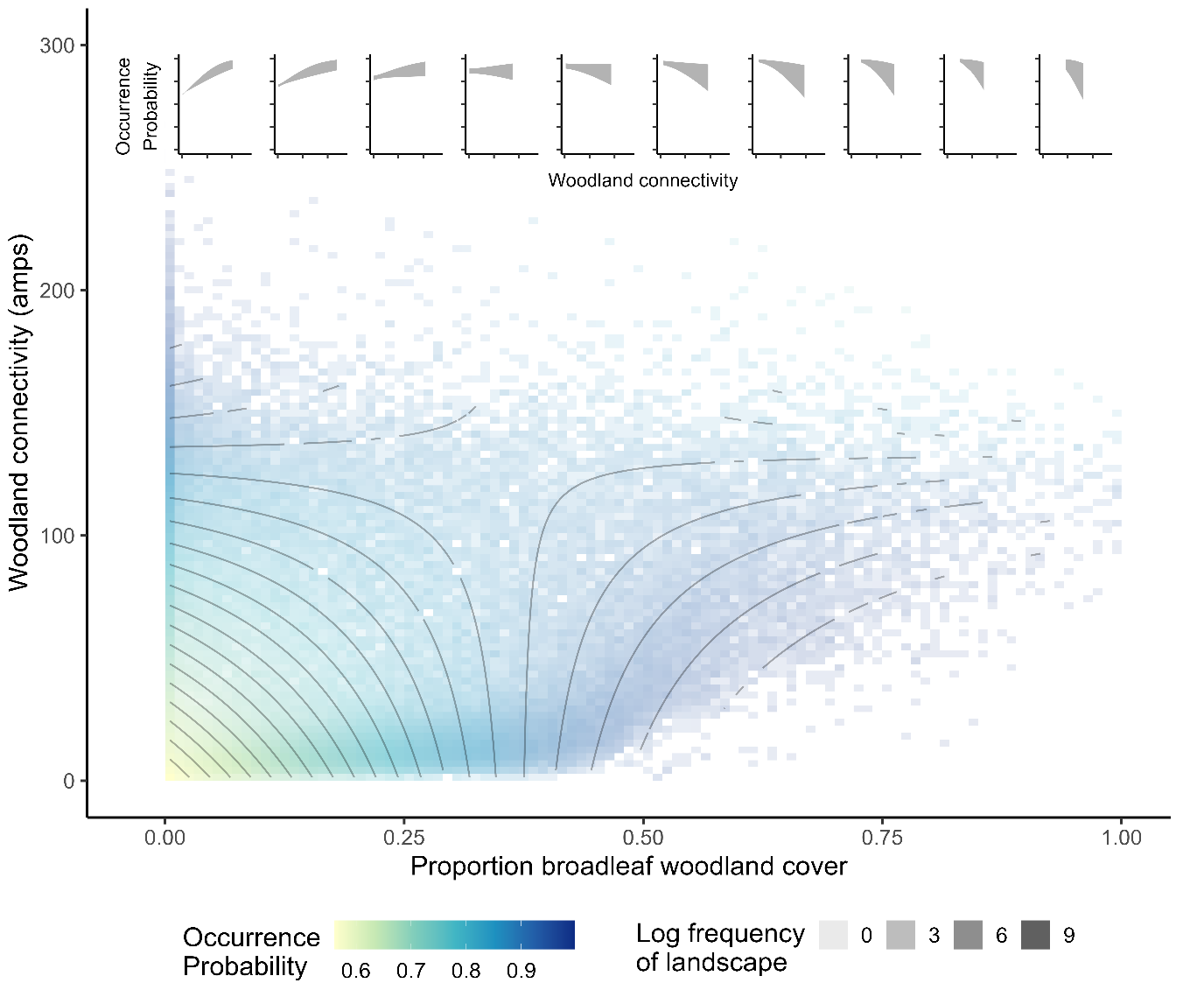


Figure S13. Interaction between broadleaf cover and woodland connectivity using estimated pooled mean effect sizes from Bayesian meta-analysis of broadleaf-associated species. Each coloured cell represents a real 1x1km cell cover-connectivity combination, with the transparency of the cell corresponding to its frequency in the UK (on a log scale) and the colour corresponding to the occurrence of species associated with broadleaf woodland. Increasingly yellow colours show that species occurrence is predicted to be lowest for low cover-low connectivity cells. Grey contour lines are added to aid visualisation with a bin width of 0.02. Smaller plots at the top of the figure show the relationship of occurrence probability to connectivity, for given fixed cover values from 0.05 to 0.95 cover in increments of 0.1, showing that woodland connectivity strongly and positively relates to the occurrence of broad-leaved associated species when broadleaved woodland cover is low, but that the relationship became negligible as the broadleaved woodland cover increases above about 25%.


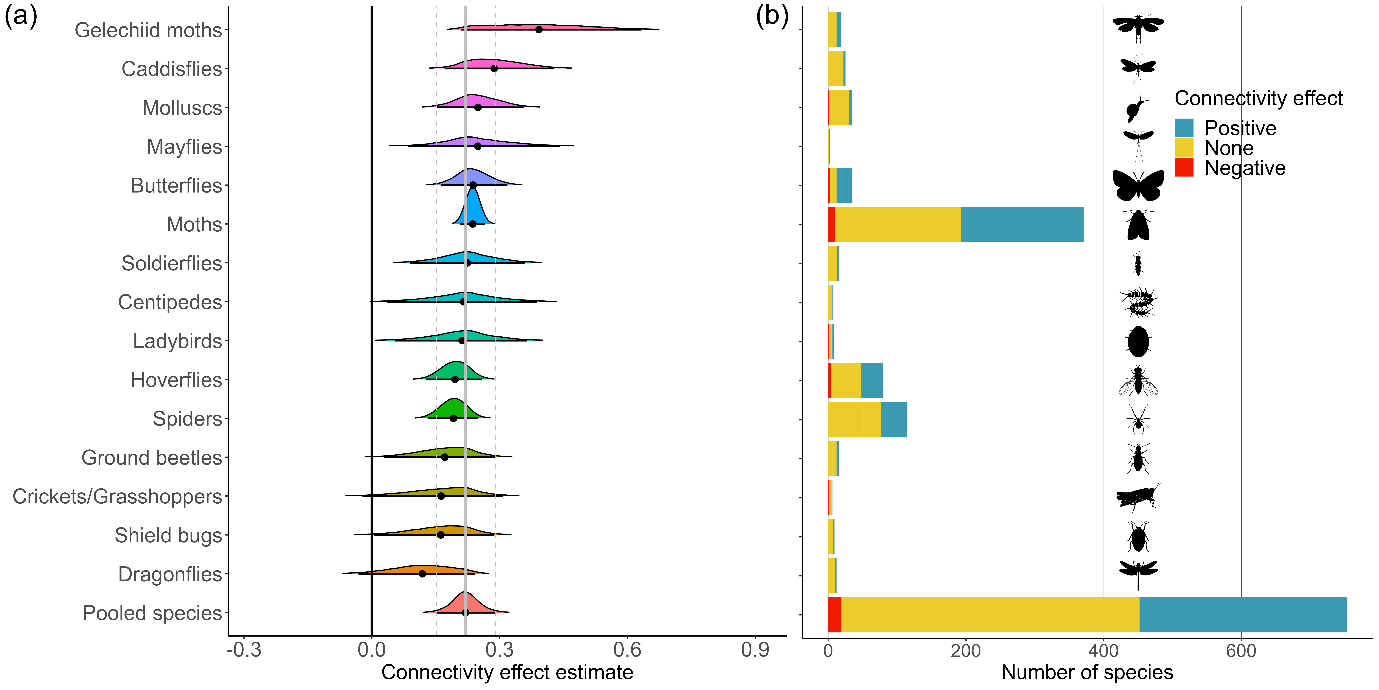


Figure S14. Connectivity effect sizes for broadleaf-associated invertebrate species, with only landscapes with broadleaf cover under 30% included. (a) Density plots of estimated effect sizes sorted by point estimates (black circles) with credible intervals (horizontal black lines) shown, and the solid and dotted grey lines indicating the estimated pooled estimate and credible interval, respectively. Spatial variables were standardised, hence effect size shown when broadleaf cover is at the mean value of 5.54%. (b) Individual species effect sizes were more often positive (credible interval entirely above zero) than negative (credible interval entirely below zero) for different recording schemes.


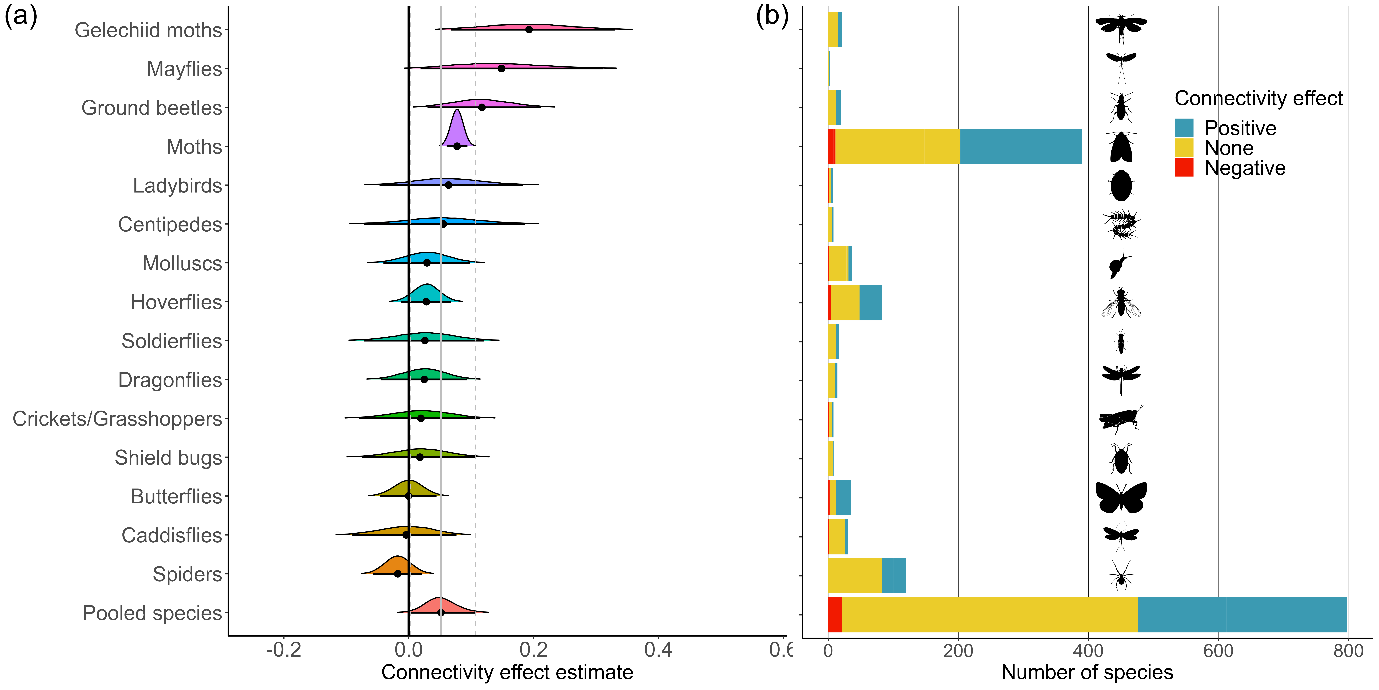
Figure S15. Connectivity effect sizes for broadleaf-associated invertebrate species, with additional broadleaf cover quadratic term included. (a) Density plots of estimated effect sizes sorted by point estimates (black circles) with credible intervals (horizontal black lines) shown, and the solid and dotted grey lines indicating the estimated pooled estimate and credible interval, respectively. Spatial variables were standardised, hence effect size shown when broadleaf cover is at the mean value of 5.54%. (b) Individual species effect sizes were more often positive (credible interval entirely above zero) than negative (credible interval entirely below zero) for different recording schemes.


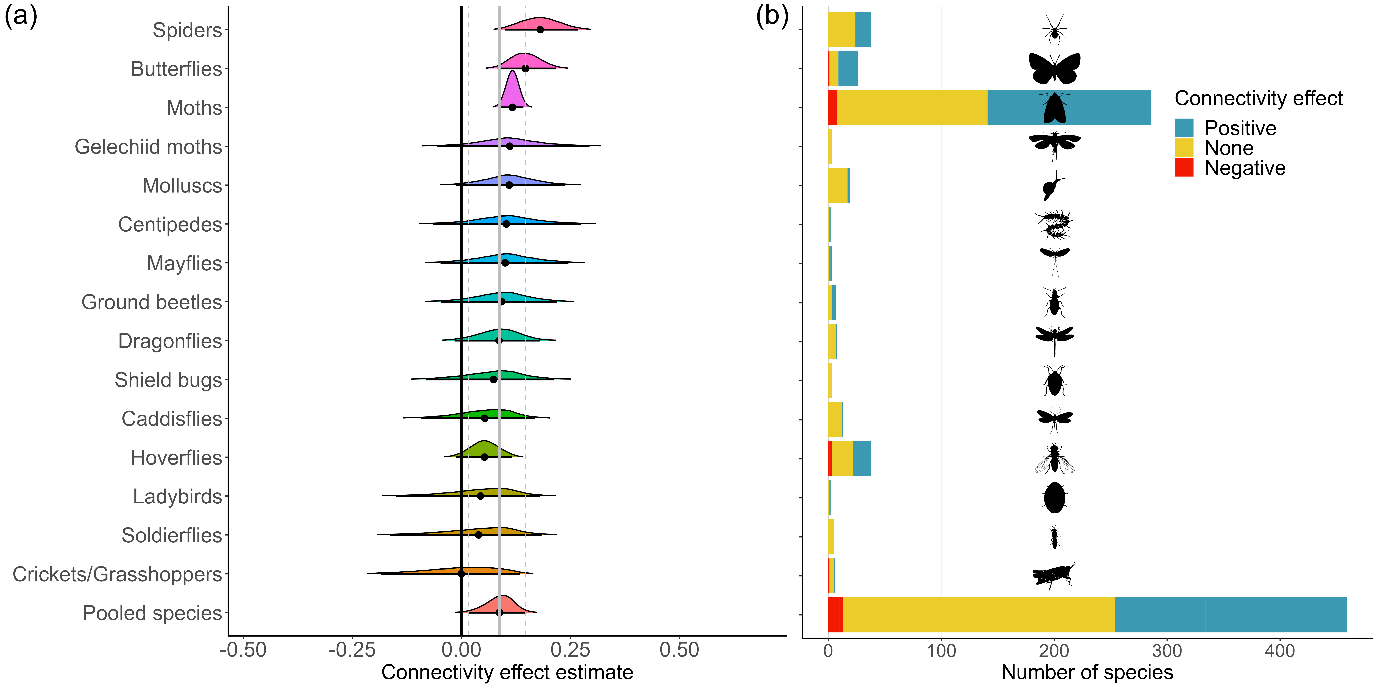


Figure S16. Connectivity effect sizes for broadleaf-associated invertebrate species, with only landscapes within 0-25% broadleaf cover quantile included (0.01-1.75% broadleaf cover). (a) Density plots of estimated effect sizes sorted by point estimates (black circles) with credible intervals (horizontal black lines) shown, and the solid and dotted grey lines indicating the estimated pooled estimate and credible interval, respectively. Spatial variables were standardised, hence effect size shown when broadleaf cover is at the mean value of 5.54%. (b) Individual species effect sizes were more often positive (credible interval entirely above zero) than negative (credible interval entirely below zero) for different recording schemes.


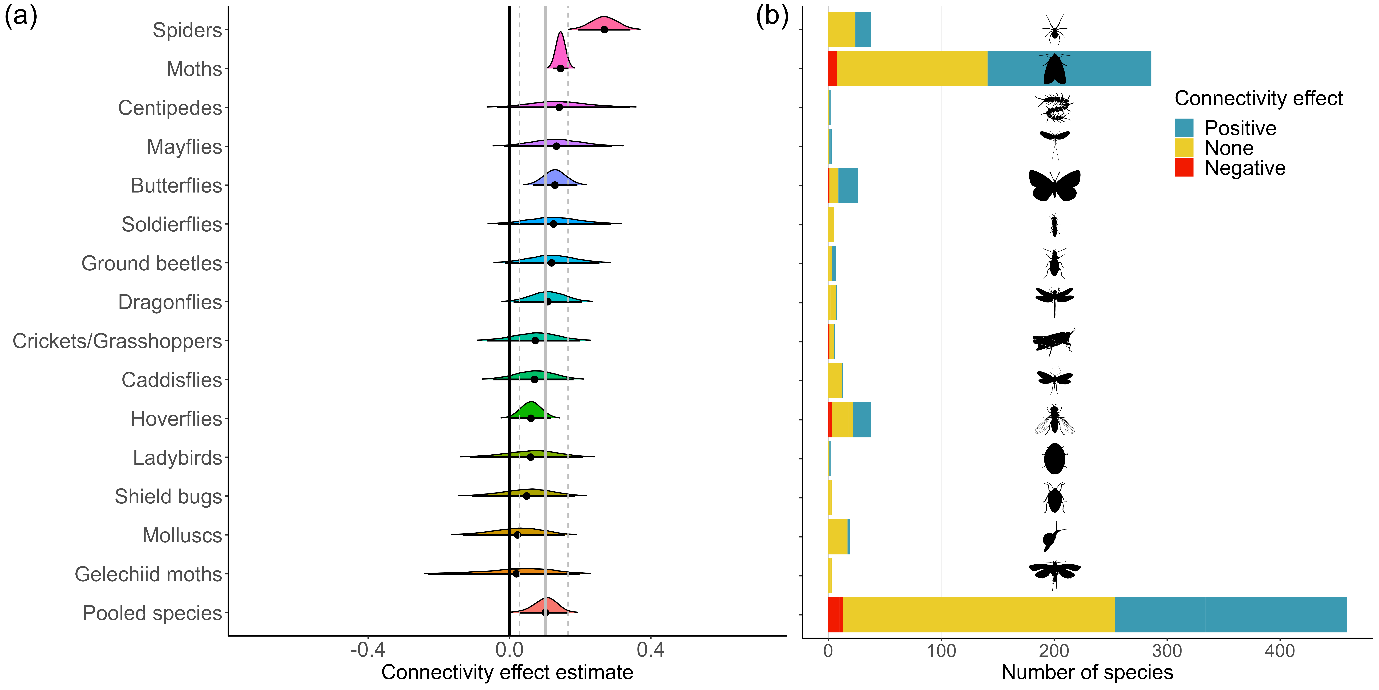


Figure S17. Connectivity effect sizes for broadleaf-associated invertebrate species, with only landscapes within 25-50% broadleaf cover quantile included (1.75-5.18% broadleaf cover). (a) Density plots of estimated effect sizes sorted by point estimates (black circles) with credible intervals (horizontal black lines) shown, and the solid and dotted grey lines indicating the estimated pooled estimate and credible interval, respectively. Spatial variables were standardised, hence effect size shown when broadleaf cover is at the mean value of 5.54%. (b) Individual species effect sizes were more often positive (credible interval entirely above zero) than negative (credible interval entirely below zero) for different recording schemes.


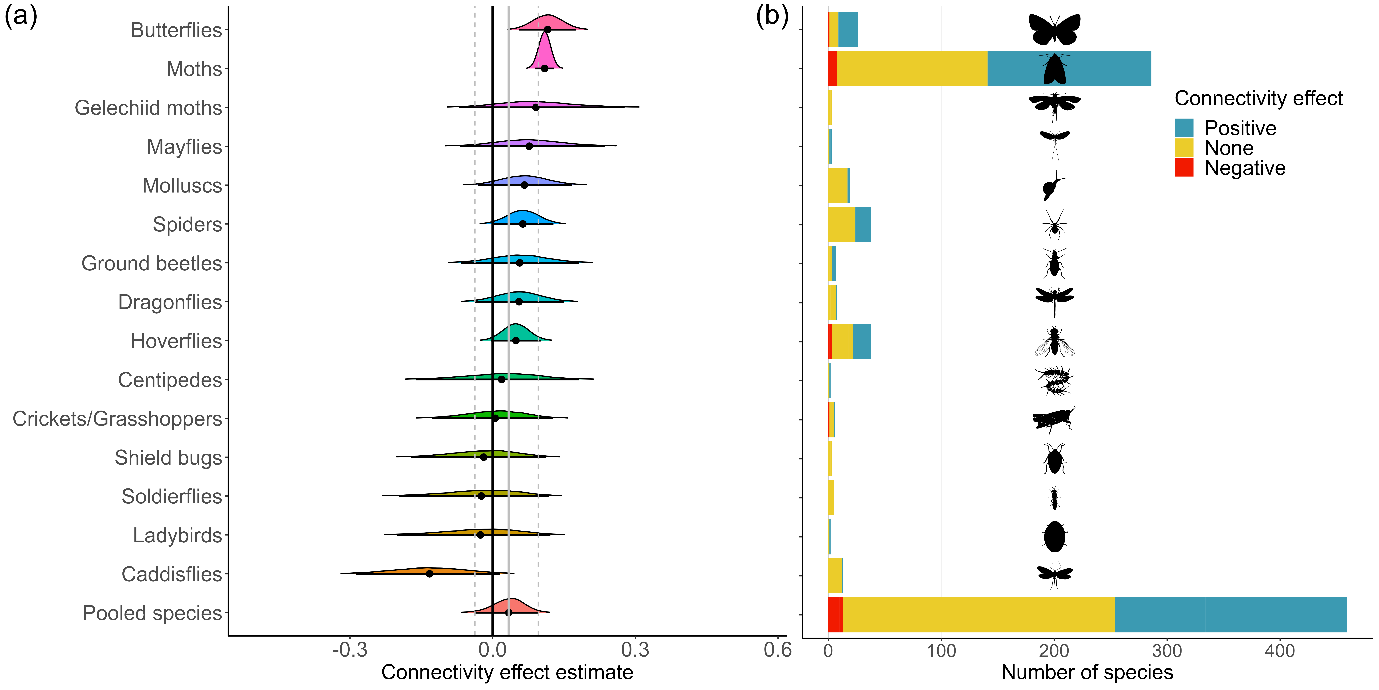


Figure S18. Connectivity effect sizes for broadleaf-associated invertebrate species, with only landscapes within 50-75% broadleaf cover quantile included (5.18-13.25% broadleaf cover). (a) Density plots of estimated effect sizes sorted by point estimates (black circles) with credible intervals (horizontal black lines) shown, and the solid and dotted grey lines indicating the estimated pooled estimate and credible interval, respectively. Spatial variables were standardised, hence effect size shown when broadleaf cover is at the mean value of 5.54%. (b) Individual species effect sizes were more often positive (credible interval entirely above zero) than negative (credible interval entirely below zero) for different recording schemes.


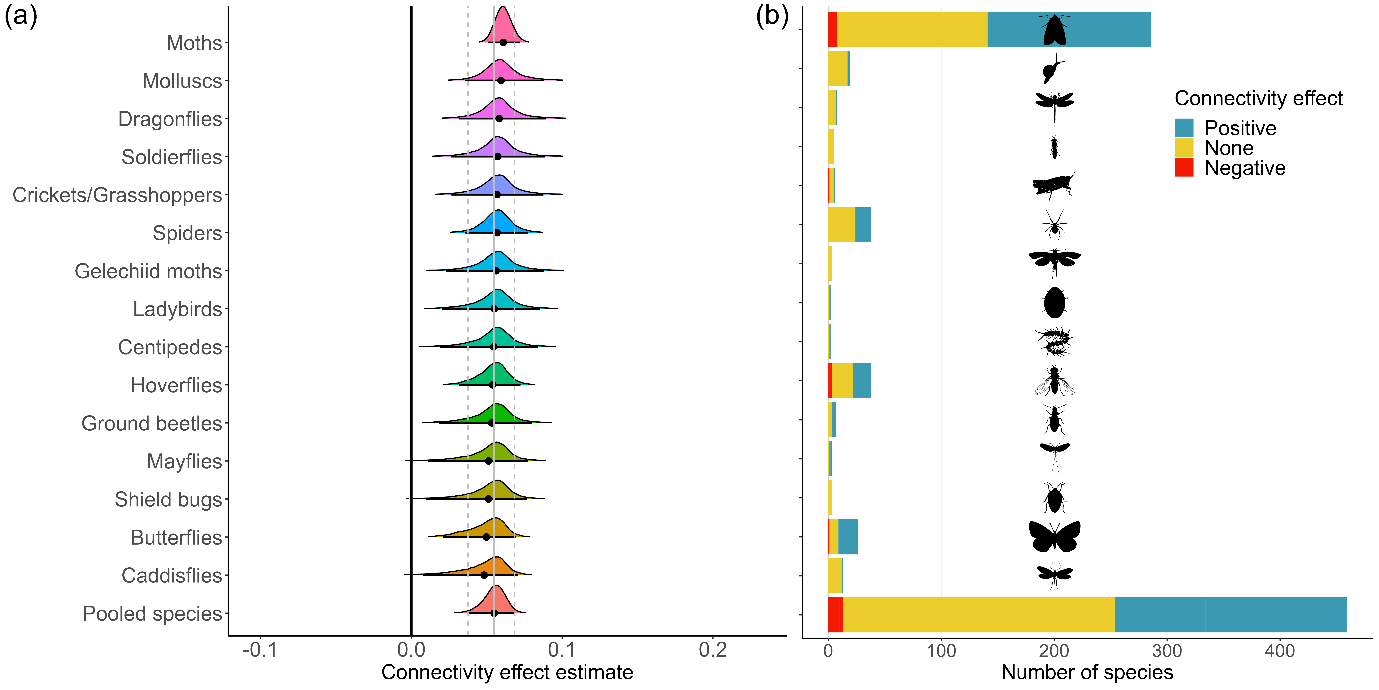


Figure S19. Connectivity effect sizes for broadleaf-associated invertebrate species, with only landscapes within 75-100% broadleaf cover quantile included (13.25-100.00%% broadleaf cover). (a) Density plots of estimated effect sizes sorted by point estimates (black circles) with credible intervals (horizontal black lines) shown, and the solid and dotted grey lines indicating the estimated pooled estimate and credible interval, respectively. Spatial variables were standardised, hence effect size shown when broadleaf cover is at the mean value of 5.54%. (b) Individual species effect sizes were more often positive (credible interval entirely above zero) than negative (credible interval entirely below zero) for different recording schemes

Table S1. Taxonomic groups and species included in the analysis. A total of 15 taxonomic groups and 3277 species were included in the initial modelling stage. For each species, records are typically collected opportunistically by volunteers and subsequently verified for plausibility by members of the respective specialist recording society, who have strong taxonomic identification skills. * All records for Northern Ireland were provided by the Centre for Environmental Data and Recording (CEDaR) with the exception of butterfly and moth data. A ‘record’ is a unique combination of species, date and 1km grid square; a ‘visit’ is one or more species from the same date and 1km grid square.

| Taxonomic group | Recording scheme* | Number of species | Total number of records | Number of filtered records in both periods | Number of filtered records in the first period | Number of filtered records in the second period | Number of visits in both periods | Number of visits in the first period | Number of visits in the second period |
| --- | --- | --- | --- | --- | --- | --- | --- | --- | --- |
| Butterflies | Butterfly Conservation | 64 | 15120259 | 6856480 | 2174581 | 4681899 | 1573388 | 497284 | 1076104 |
| Caddisflies | Trichoptera (caddisfly) Recording Scheme | 186 | 328798 | 195648 | 81394 | 114254 | 59772 | 29433 | 30339 |
| Carabids | Ground Beetle Recording Scheme | 371 | 440519 | 120360 | 85675 | 34685 | 22290 | 10285 | 12005 |
| Centipedes | Centipede Recording Scheme | 45 | 50004 | 13402 | 10301 | 3101 | 5916 | 4031 | 1885 |
| Ephemeroptera | Ephemeroptera (mayfly) Recording Scheme | 54 | 339391 | 169778 | 101069 | 68709 | 60837 | 38912 | 21925 |
| Gelechiidae | Gelechiid Recording Scheme | 151 | 115402 | 54807 | 12831 | 41976 | 35141 | 8240 | 26901 |
| Hoverflies | Hoverfly Recording Scheme | 262 | 1240514 | 733962 | 168326 | 565636 | 206953 | 31383 | 175570 |
| Ladybirds | UK Ladybird Survey | 53 | 325390 | 132334 | 25421 | 106913 | 81471 | 11184 | 70287 |
| Molluscs | The Conchological Society of Great Britain and Ireland | 208 | 256975 | 86402 | 39779 | 46623 | 19437 | 5236 | 14201 |
| Moths | Butterfly Conservation | 864 | 28218310 | 8692842 | 2630743 | 6062099 | 892541 | 332664 | 559877 |
| Odonata | British Dragonfly Society | 51 | 1061119 | 459544 | 194539 | 265005 | 134874 | 46023 | 88851 |
| Orthoptera | Orthoptera Recording Scheme | 65 | 270124 | 132271 | 39938 | 92333 | 50431 | 16855 | 33576 |
| Shieldbugs | Terrestrial Heteroptera Recording Scheme | 75 | 138993 | 80351 | 5127 | 75224 | 52720 | 3042 | 49678 |
| Soldierflies | Soldierflies and Allies Recording Scheme | 154 | 143505 | 65855 | 18504 | 47351 | 38314 | 7097 | 31217 |
| Spiders | Spider Recording Scheme | 674 | 894849 | 444887 | 319006 | 125881 | 48097 | 32062 | 16035 |
|  |  | 3277 | 48944152 | 18238923 | 5907234 | 12331689 | 3282182 | 1073731 | 2208451 |
